# Supplementary material for: Anthraquinones, Diphenyl Ethers, and Their Derivatives from the Culture of the Marine Sponge-Associated Fungus Neosartorya spinosa KUFA 1047
Source: Mar Drugs. 2021 Aug 11;19(8):457. doi: 10.3390/md19080457 (PMC8401666; doi:10.3390/md19080457)
Supplement: Supplementary file 1 [file marinedrugs-19-00457-s001.zip › marinedrugs-1327307-supplementary.pdf]

Supplementary Materials

Anthraquinones, Diphenyl Ethers and Their Derivatives from the Culture of the Marine Sponge-Associated Fungus *Neosartorya spinosa* KUFA 1047

Joana D. M. de Sá [1], José A. Pereira [2, 3], Tida Dethoup [4], Honorina Cidade [1, 3], Maria Emília Sousa[1,3], Inês C. Rodrigues [2], Paulo M. Costa [2, 3], Sharad Mistry [5], Artur M. S. Silva [6], Anake Kijjoa [2,3*]

[1] Laboratório de Química Orgânica, Departamento de Ciências Químicas, Faculdade de Farmácia, Universidade do Porto, Rua de Jorge Viterbo Ferreira, 228, 4050-313 Porto, Portugal. E-mail: joanadmsa2703@gmail.com (J.D. M. S.), hcidade@ff.up.pt (H.C.)

[2] *ICBAS*-Instituto de Ciências Biomédicas Abel Salazar, Rua de Jorge Viterbo Ferreira, 228, 4050-313 Porto, Portugal. E-mail: jpereira@icbas.up.pt (J. A. P.), inescoutorodrigues@gmail.com (I. R), pmcosta@icbas.up.pt (P.M.C.)

[3] Interdisciplinary Centre of Marine and Environmental Research (CIIMAR), Terminal de Cruzeiros do Porto de Lexões, Av. General Norton de Matos s/n, 4450-208, Matosinhos, Portugal.

[4] Department of Plant Pathology, Faculty of Agriculture, Kasetsart University, Bangkok 10240, Thailand. E-mail: tdethoup@yahoo.com

[5] Department of Chemistry, University of Leicester, University Road, Leicester LE 7RH, UK, E-mail: scm11@leicester.ac.uk

[6] Departamento de Química & QOPNA, Universidade de Aveiro, 3810-193 Aveiro, Portugal. E-mail: artur.silva@ua.pt

*Correspondence: ankijjoa@icbas.up.pt; Tel. +351-22-042-8331; Fax: + 351-22-206-2232

Figure S1. ^{1}H NMR spectrum of **1** (DMSO-d_6, 300MHz).

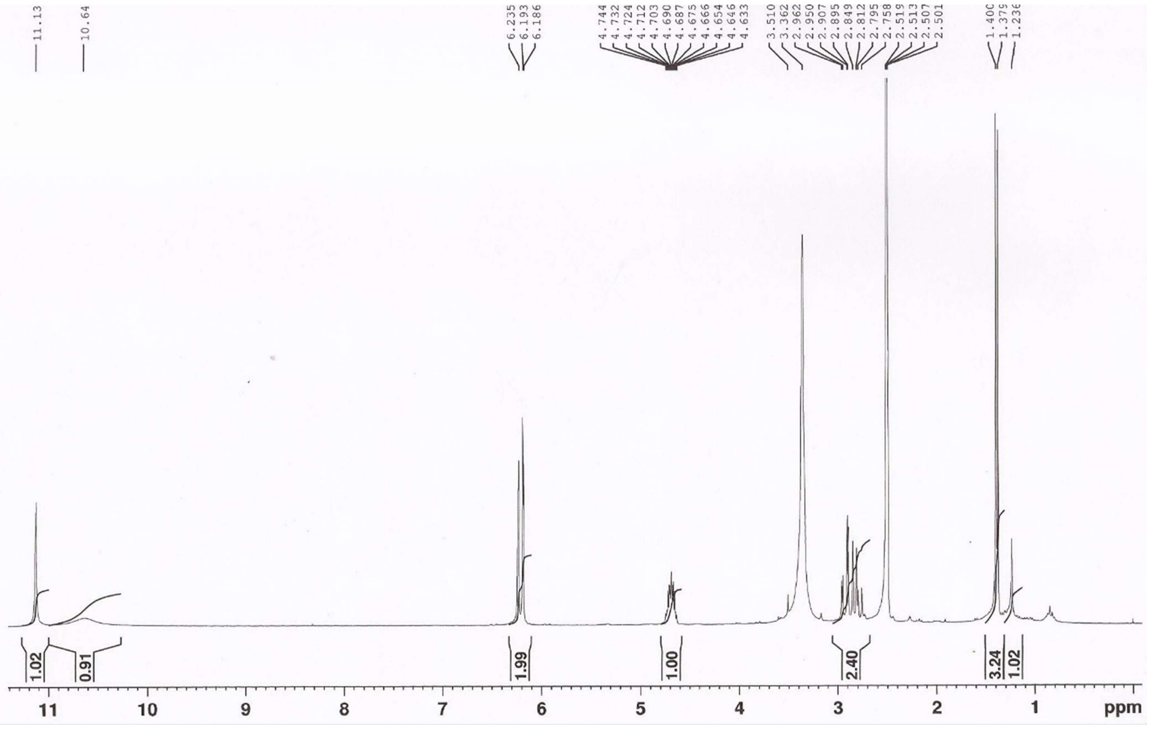

Figure S2. ^{13}C NMR spectrum of **1** (DMSO-d_6, 75 MHz).

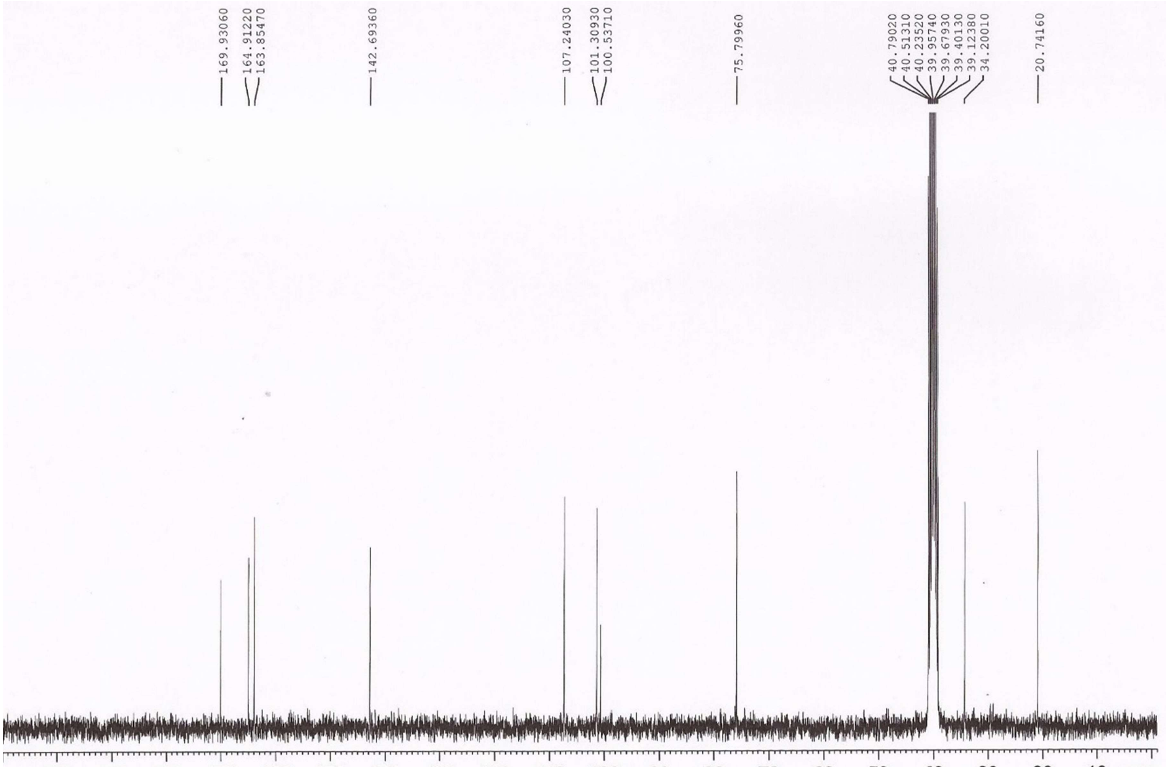

Figure S3. COSY spectrum of **1** (DMSO-d_6, 300 MHz).

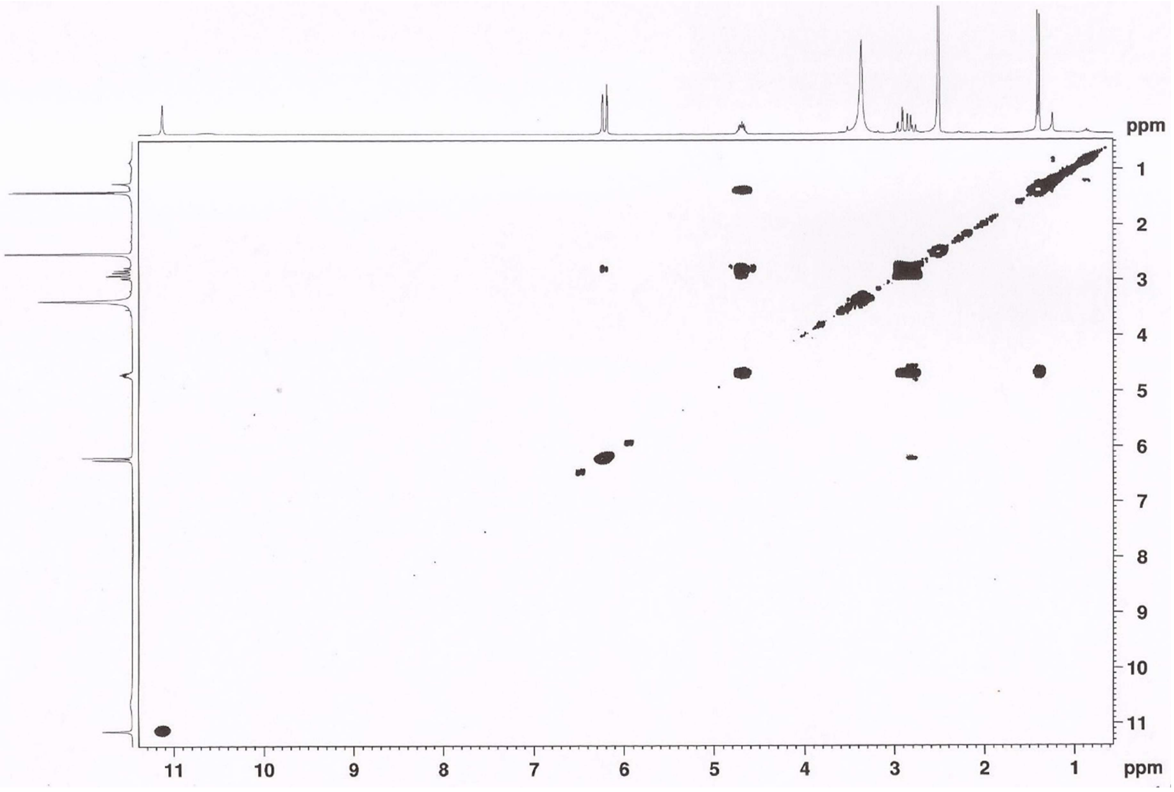

Figure S4. HSQC spectrum of **1** (DMSO-d_6, 300 MHz).

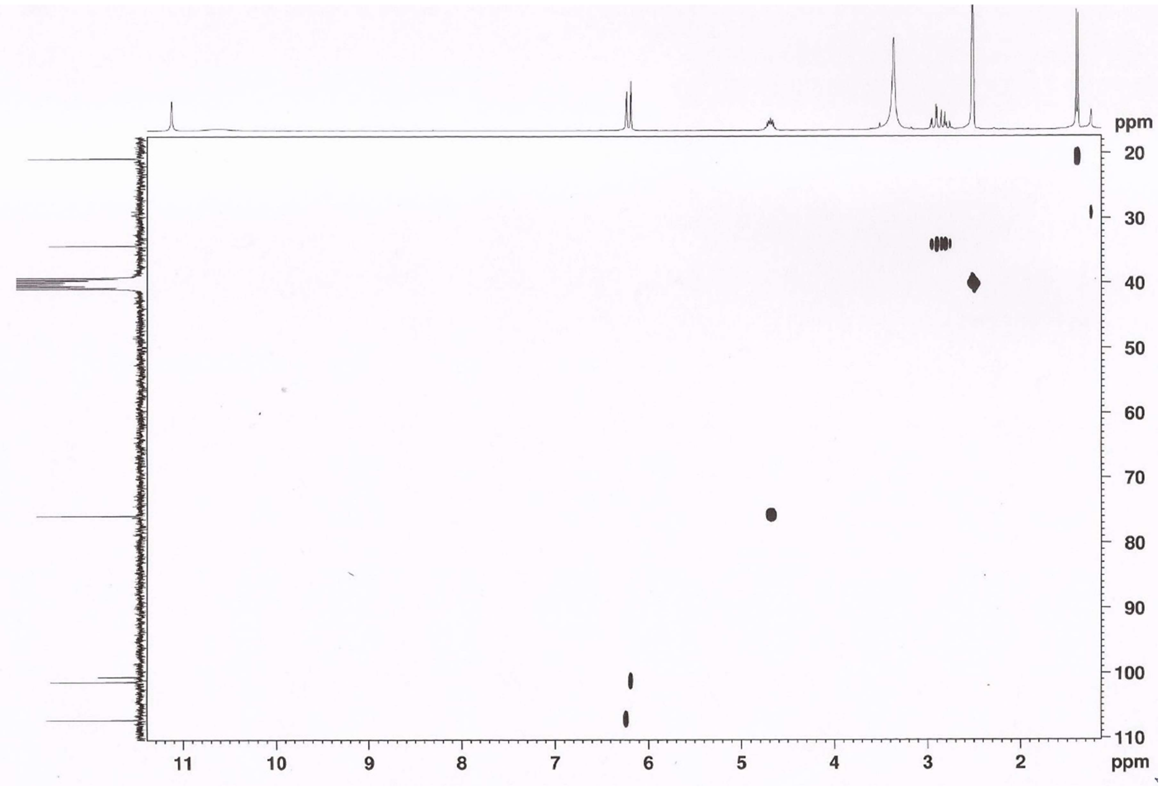

Figure S5. HMBC spectrum of **1** (DMSO-d_6, 300 MHz).

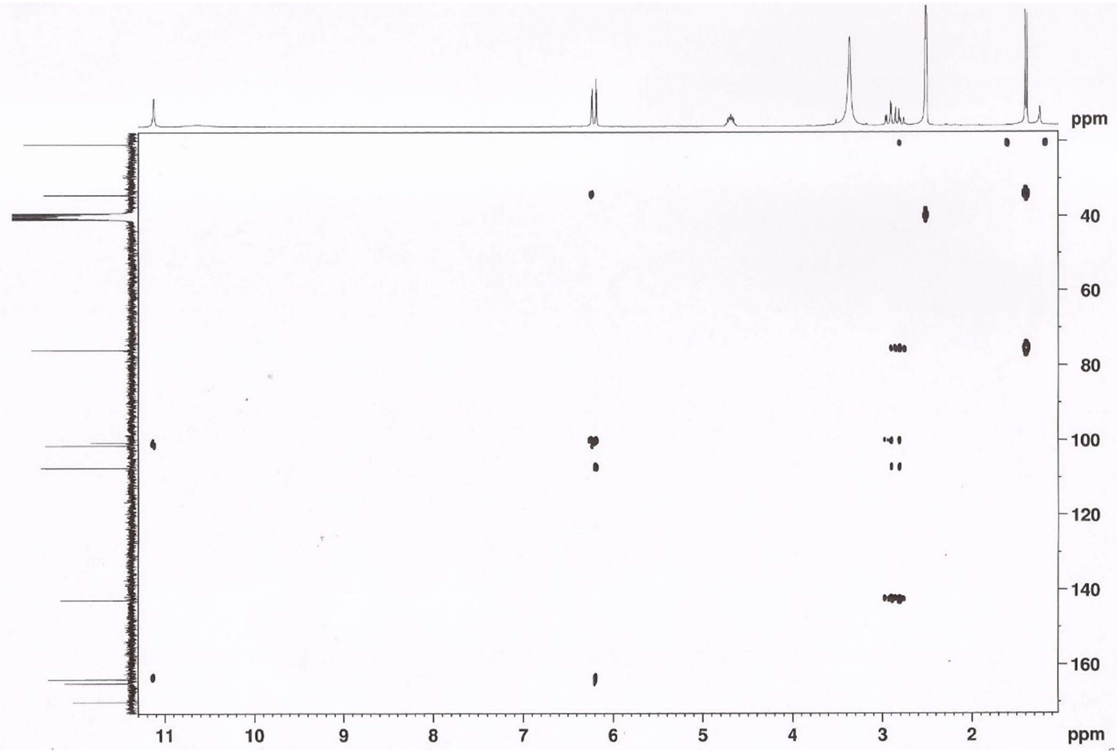

Figure S6. (+)-HRESIMS of **1**.

Single Mass Analysis
Tolerance = 5.0 PPM / DBE: min = -1.5, max = 100.0
Element prediction: Off
Number of isotope peaks used for i-FIT = 3

Monoisotopic Mass, Even Electron Ions
12 formula(e) evaluated with 1 results within limits (up to 100 best isotopic matches for each mass)
Elements Used:
C: 10-10 H: 0-150 O: 0-50

Minimum:				-1.5				
Maximum:		5.0	5.0	100.0				
Mass	Calc. Mass	mDa	PPM	DBE	i-FIT	Norm	Conf(%)	Formula
195.0657	195.0657	0.0	0.0	5.5	515.3	n/a	n/a	C10 H11 O4

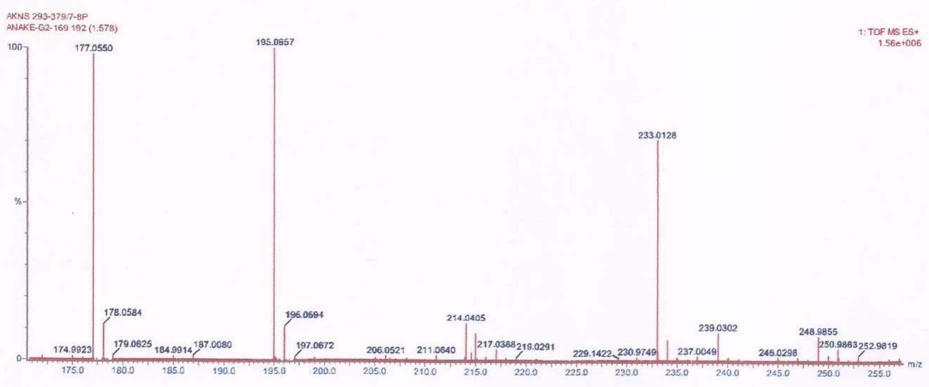

Figure S7. ^{1}H NMR spectrum of **2** (DMSO-d_6, 300MHz).

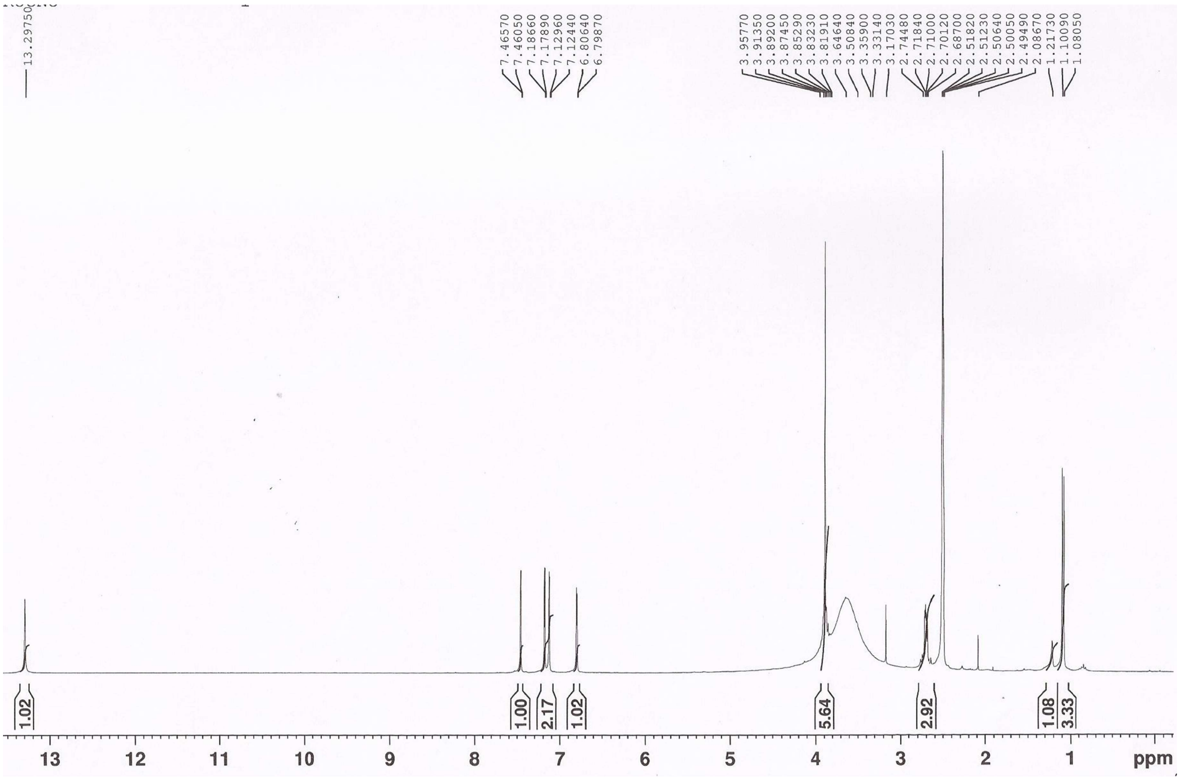

Figure S8. ^{13}C NMR spectrum of **2** (DMSO-d_6, 75MHz).

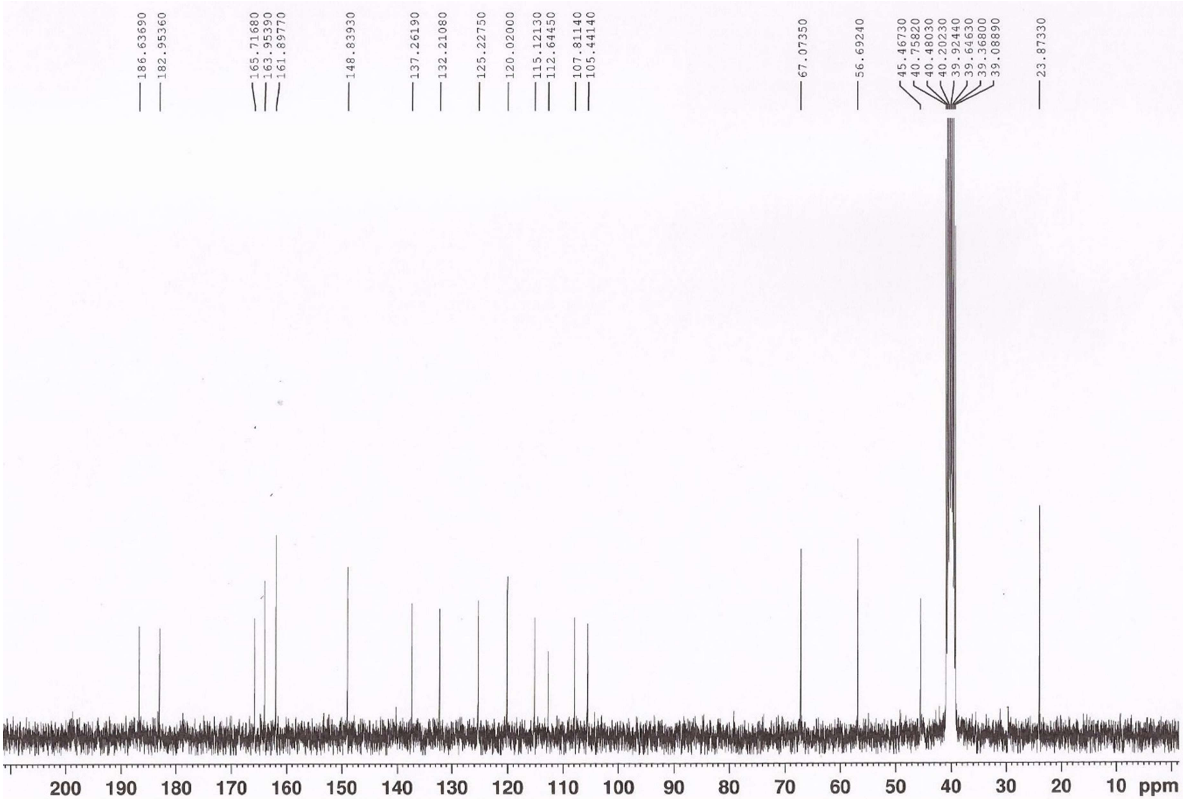

Figure S9. COSY spectrum of **2** (DMSO-d_6, 300 MHz).

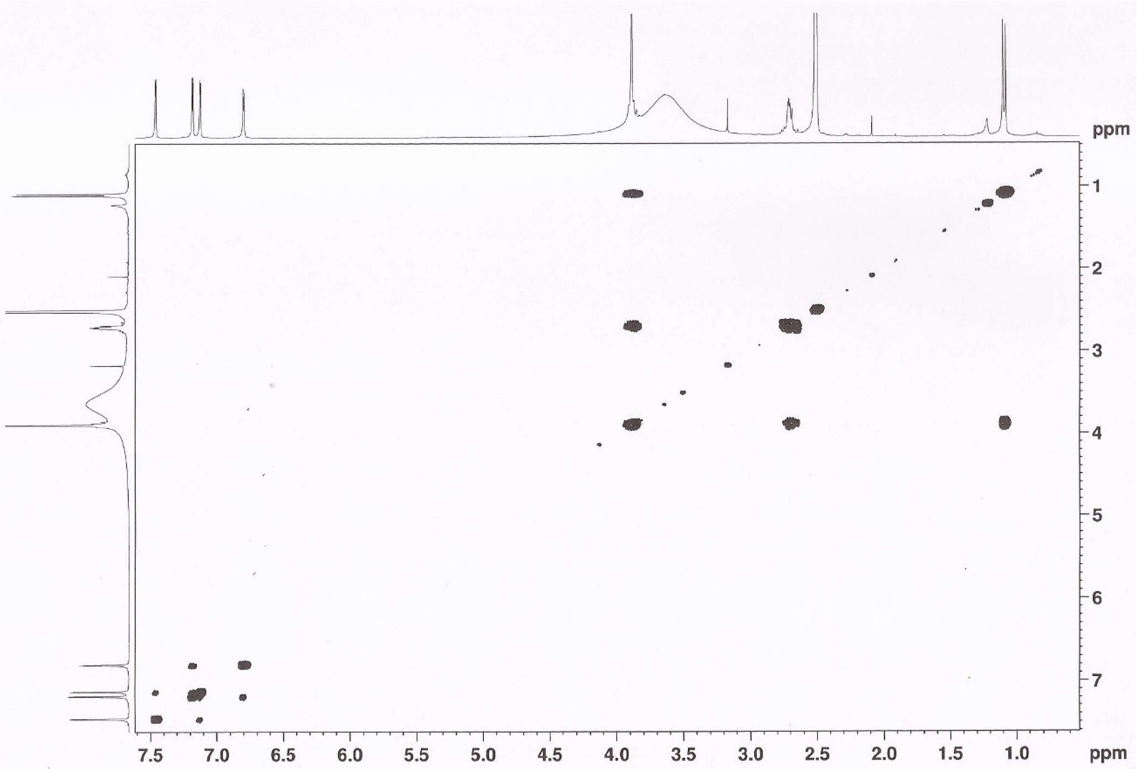

Figure S10. HSQC spectrum of **2** (DMSO-d_6, 300 MHz).

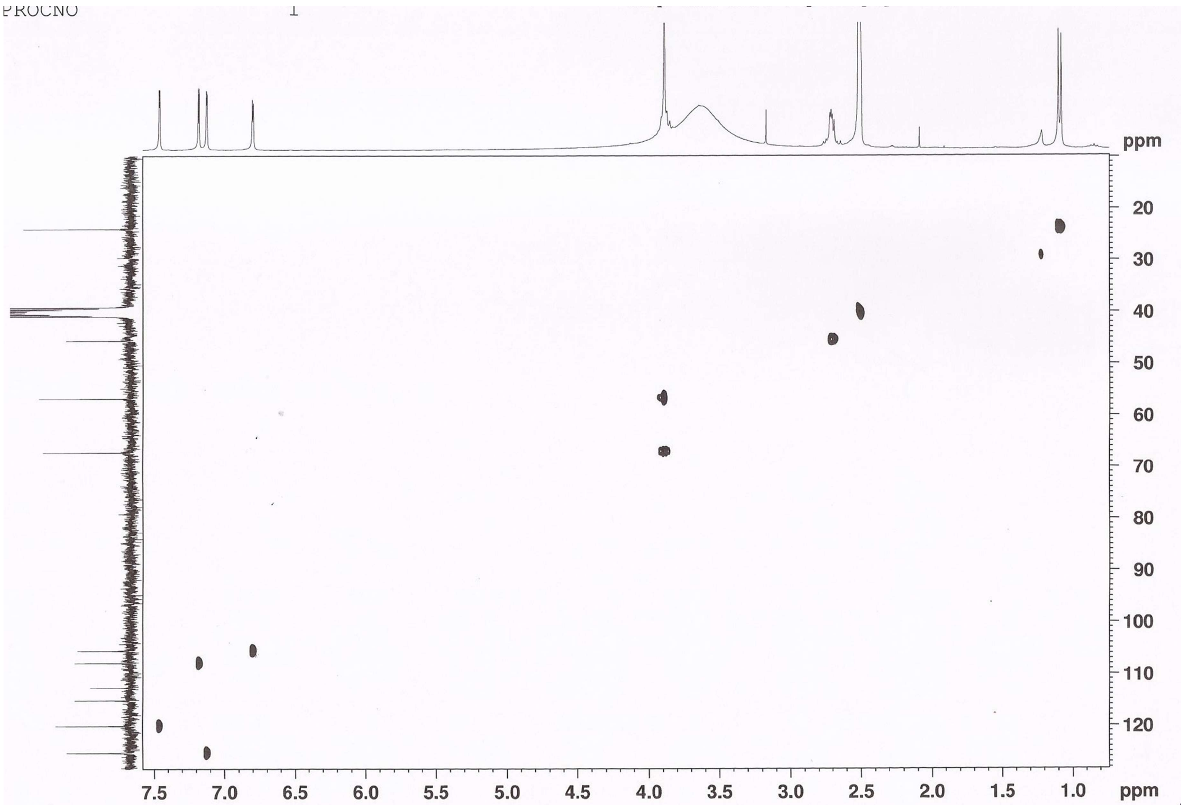

Figure S11. HMBC spectrum of **2** (DMSO-d_6, 300 MHz).

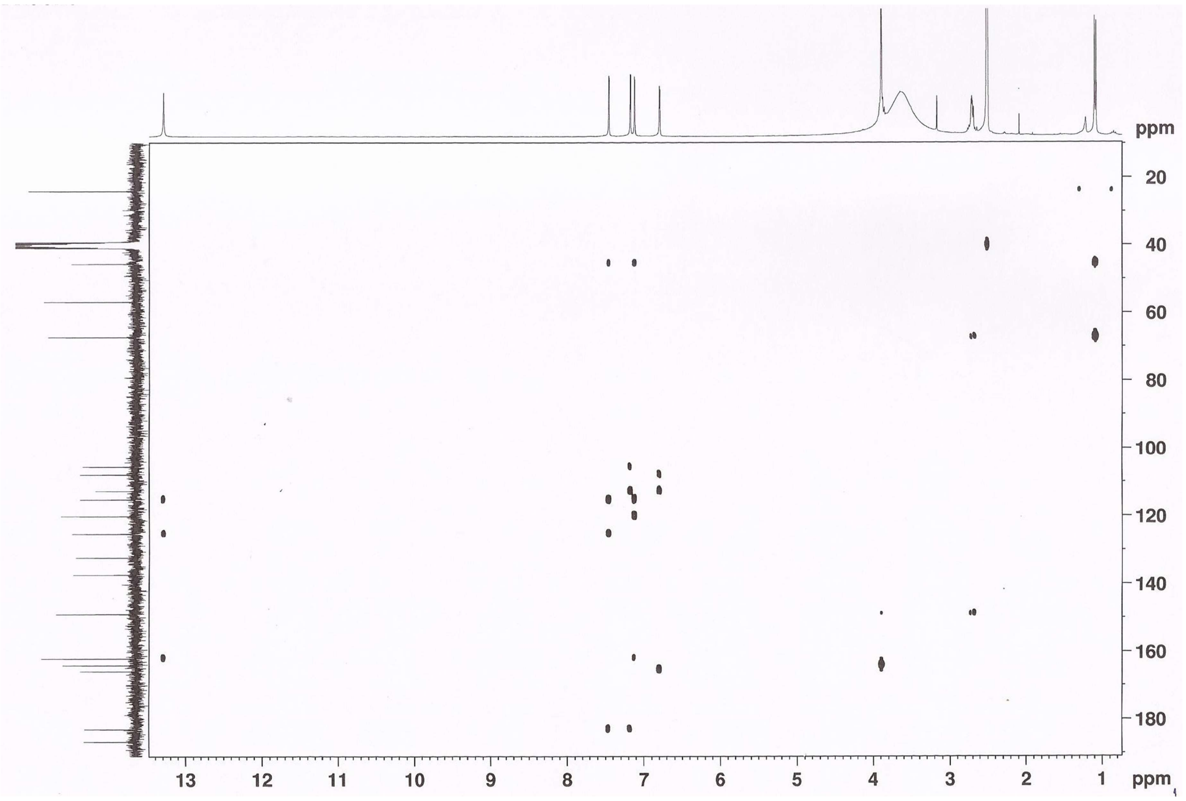

Figure S12. (+)-HRESIMS of **2**.

Elemental Composition Report [MH]+

Single Mass Analysis
Tolerance = 5.0 PPM / DBE: min = -1.5, max = 50.0
Element prediction: Off
Number of isotope peaks used for i-FIT = 3

Monoisotopic Mass, Even Electron Ions
21 formula(e) evaluated with 1 results within limits (up to 100 best isotopic matches for each mass)
Elements Used:
C: 18-18 H: 0-150 O: 0-30

Minimum:				-1.5				
Maximum:		5.0	5.0	50.0				
Mass	Calc. Mass	mDa	PPM	DBE	i-FIT	Norm	Conf(%)	Formula
329.1027	329.1025	0.2	0.6	10.5	704.5	n/a	n/a	C18 H17 O6

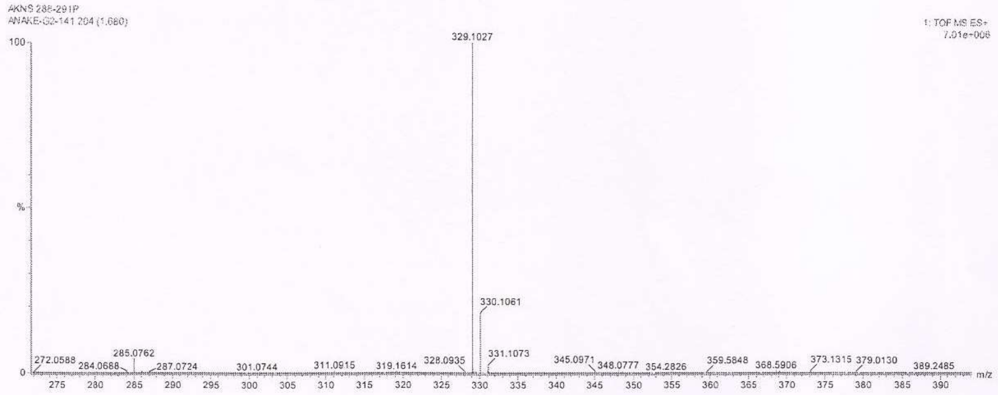

Figure S13. 1H NMR spectrum of a mixture of **3** and **4** (DMSO-d_6, 500 MHz).

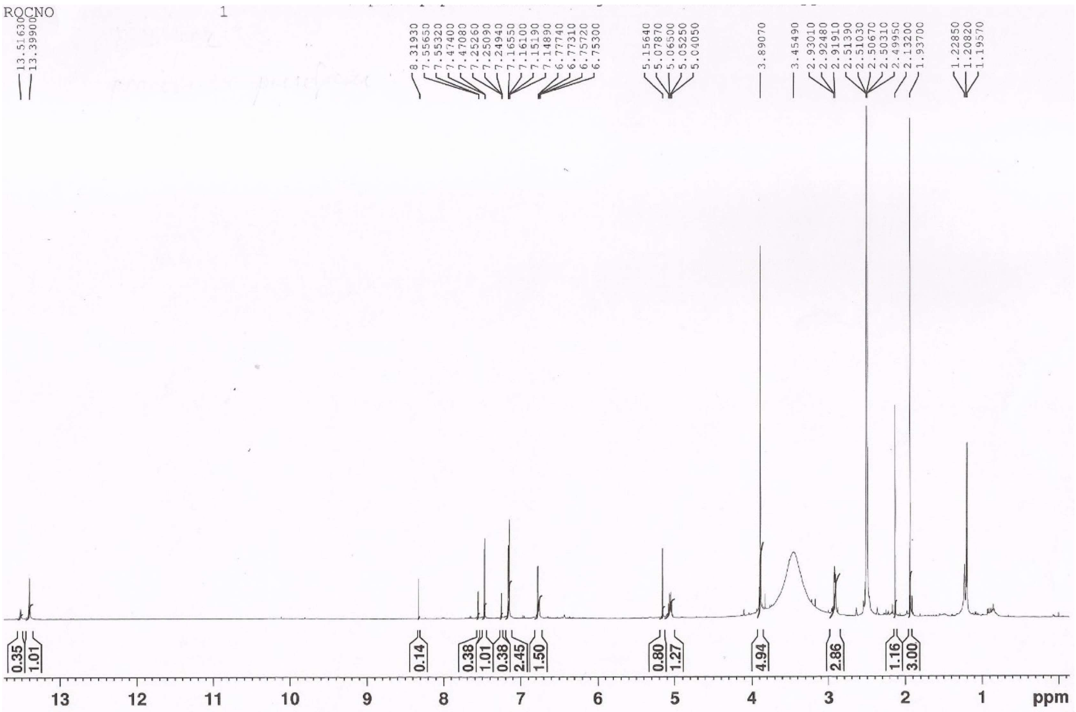

Figure S14. ^{13}C NMR spectrum of a mixture of **3** and **4** (DMSO-d_6, 125 MHz).

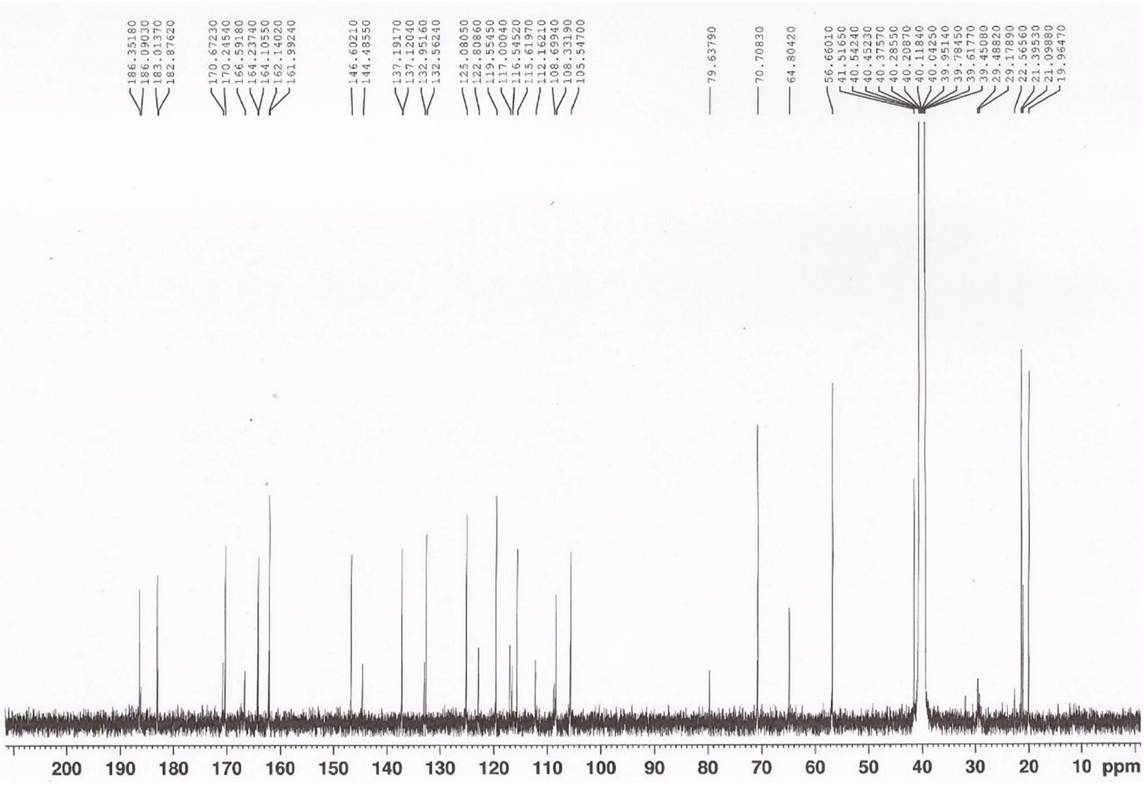

Figure S15. COSY spectrum of a mixture of **3** and **4** (DMSO-d_6, 500 MHz).

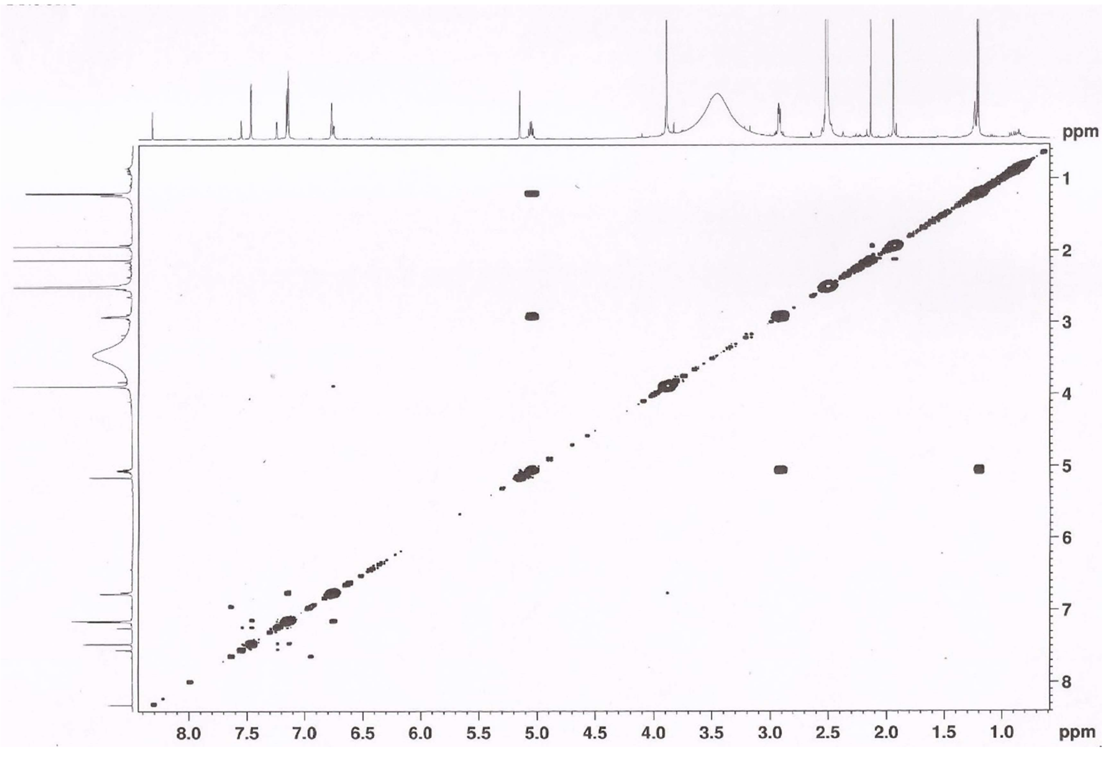

Figure S16. HSQC of a mixture of **3** and **4** (DMSO-d_6, 500 MHz).

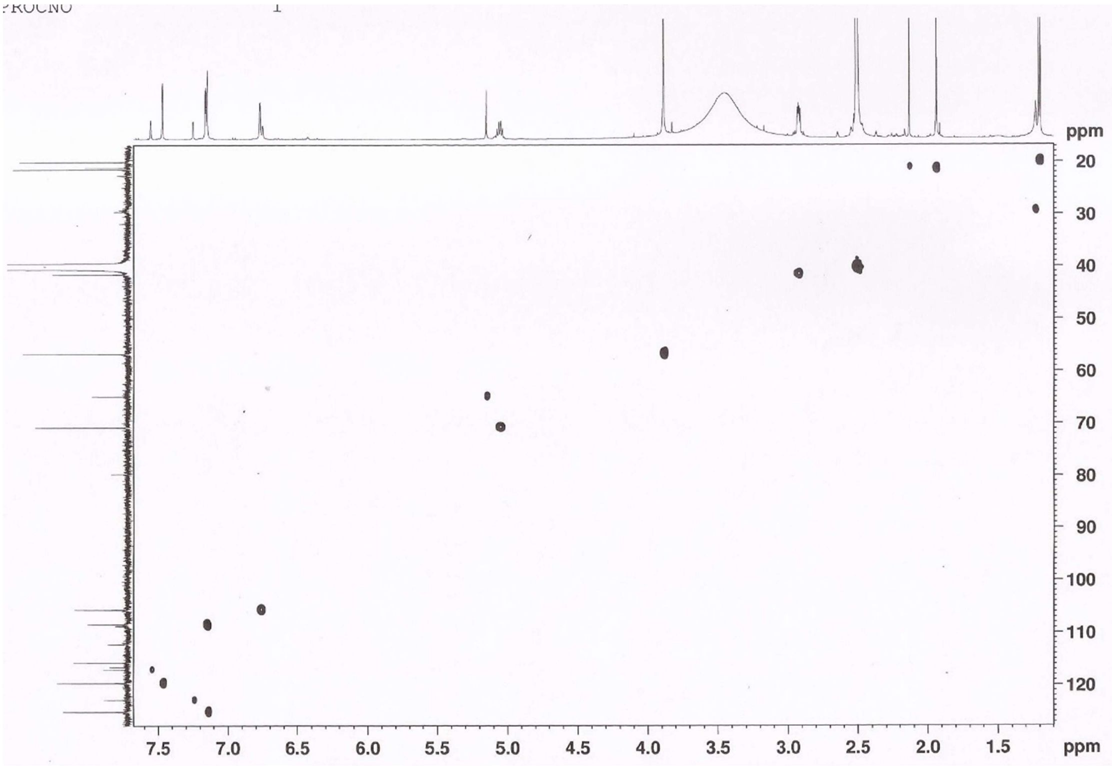

Figure S17. HMBC of a mixture of **3** and **4** (DMSO-d_6, 500 MHz).

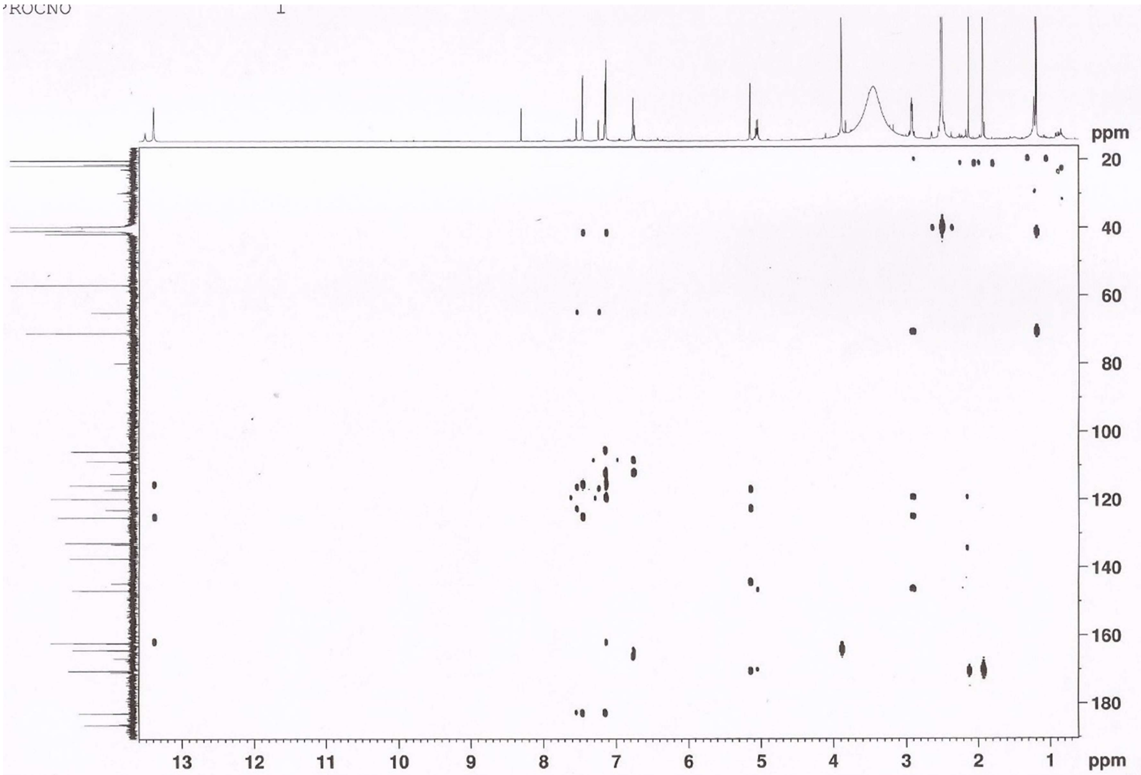

Figure S18. (+)-HRESIMS of **3**.

Elemental Composition Report [MH]⁺

Single Mass Analysis
Tolerance = 5.0 PPM / DBE: min = -1.5, max = 50.0
Element prediction: Off
Number of isotope peaks used for i-FIT = 3
Monoisotopic Mass, Even Electron Ions
22 formula(e) evaluated with 1 results within limits (up to 100 best isotopic matches for each mass)
Elements Used:
C: 18-18 H: 0-150 O: 0-30

Minimum:				-1.5				
Maximum:		5.0	5.0	50.0				
Mass	Calc. Mass	mDa	PPM	DBE	i-FIT	Norm	Conf(%)	Formula
343.0809	343.0818	-0.9	-2.6	11.5	908.8	n/a	n/a	C18 H15 O7

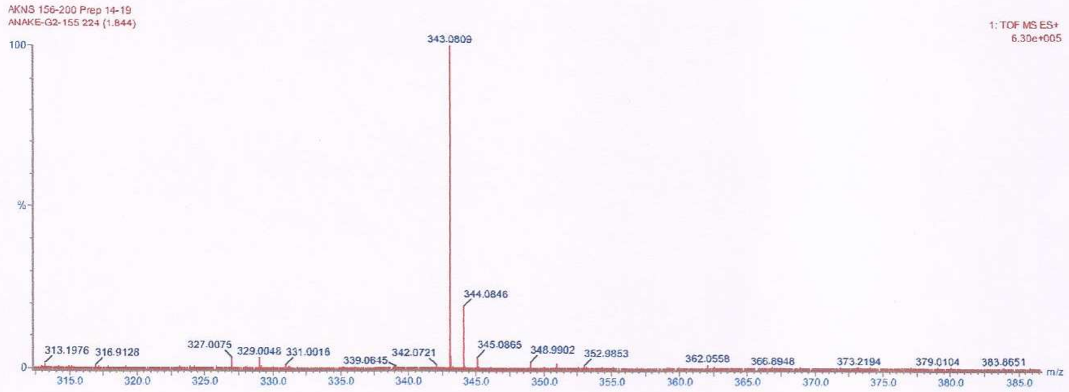

Figure S19. (+)-HRESIMS of **4**.

Elemental Composition Report [MH]+

Single Mass Analysis

Tolerance = 5.0 PPM / DBE: min = -1.5, max = 50.0

Element prediction: Off

Number of isotope peaks used for i-FIT = 3

Monoisotopic Mass, Even Electron Ions

24 formula(e) evaluated with 1 results within limits (up to 100 best isotopic matches for each mass)

Elements Used:

C: 20-20 H: 0-150 O: 0-30

Minimum:				-1.5				
Maximum:		5.0	5.0	50.0				
Mass	Calc. Mass	mDa	PPM	DBE	i-FIT	Norm	Conf(%)	Formula
371.1124	371.1131	-0.7	-1.9	11.5	480.6	n/a	n/a	C20 H19 O7

Elemental Composition Report [MNa]+

Single Mass Analysis

Tolerance = 5.0 PPM / DBE: min = -1.5, max = 50.0

Element prediction: Off

Number of isotope peaks used for i-FIT = 3

Monoisotopic Mass, Even Electron Ions

49 formula(e) evaluated with 1 results within limits (up to 100 best isotopic matches for each mass)

Elements Used:

C: 20-20 H: 0-150 O: 0-30 Na: 0-1

Minimum:				-1.5				
Maximum:		5.0	5.0	50.0				
Mass	Calc. Mass	mDa	PPM	DBE	i-FIT	Norm	Conf(%)	Formula
393.0945	393.0950	-0.5	-1.3	11.5	747.8	n/a	n/a	C20 H18 O7 Na

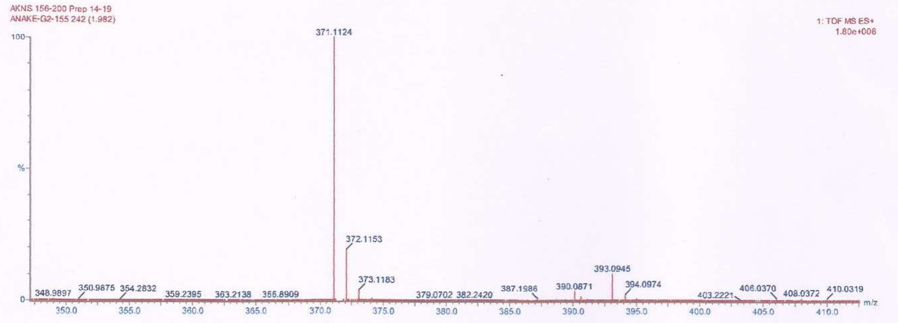

Figure S20. ^{1}H NMR spectrum of **5** (DMSO-d_6, 300 MHz).

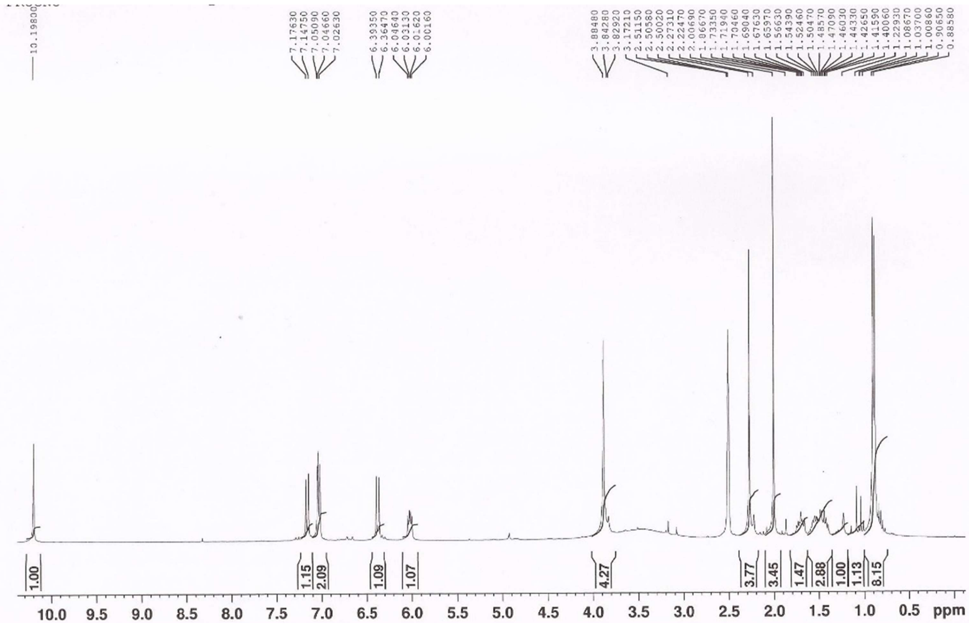

Figure S21. ^{13}C NMR spectrum of **5** (DMSO-d_6, 575 MHz).

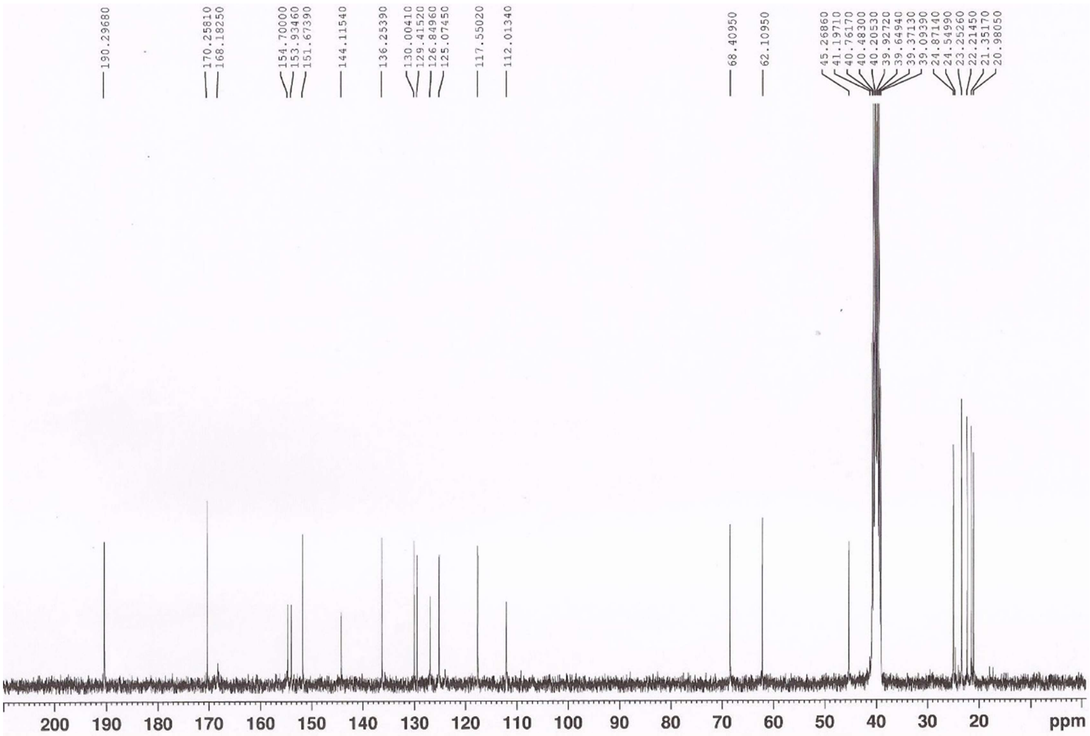

Figure S22. COSY spectrum of **5** (DMSO-d_6, 300 MHz).

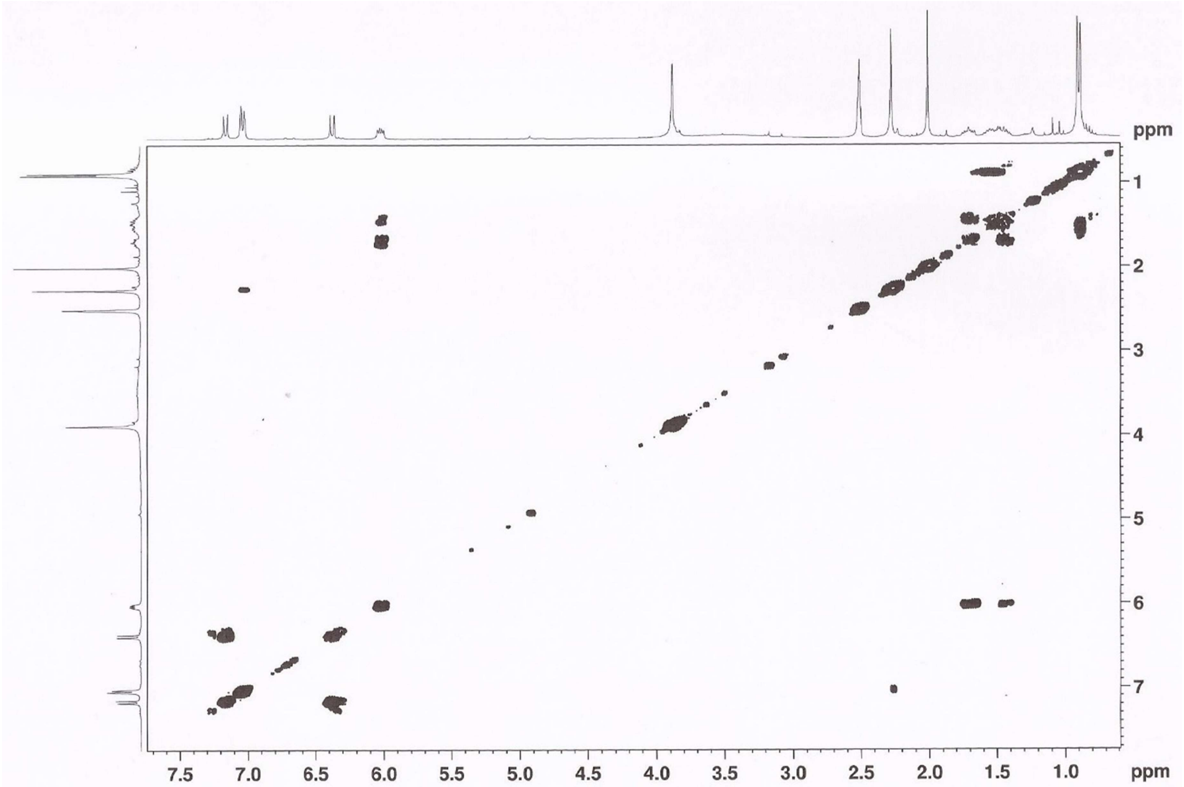

Figure S23. HSQC spectrum of **5** (DMSO-d_6, 300 MHz).

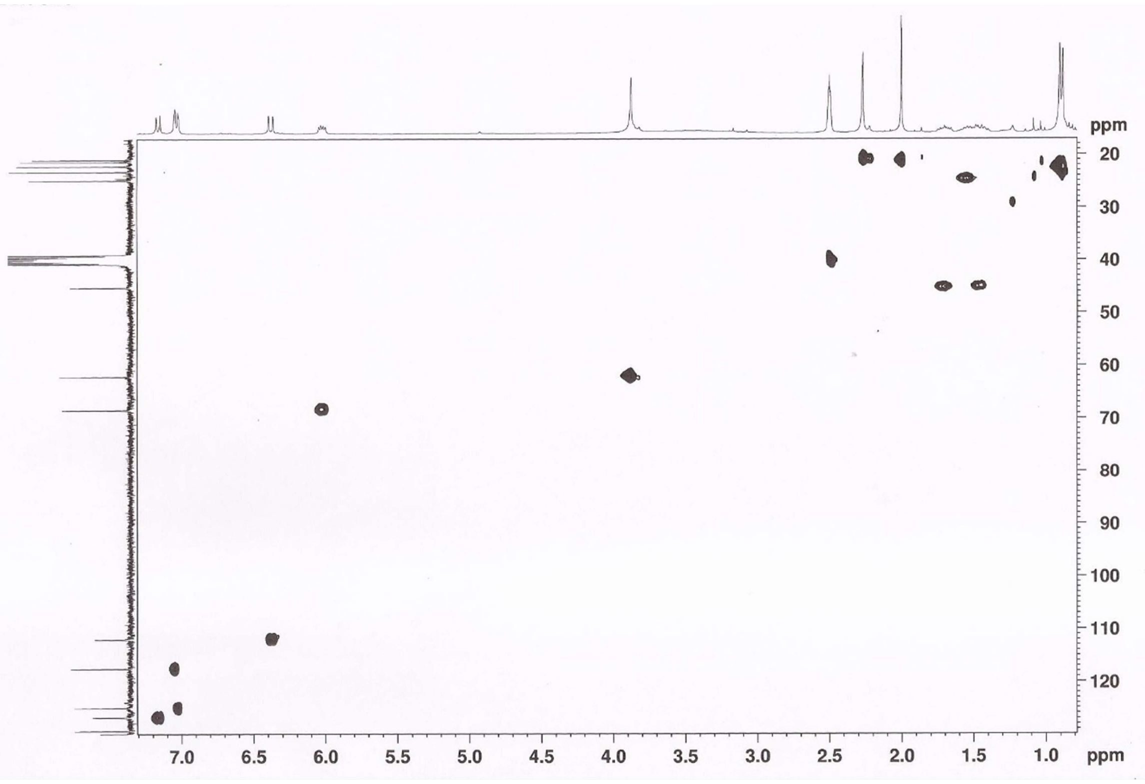

Figure S24.HMBC spectrum of **5** (DMSO-d_6, 300 MHz).

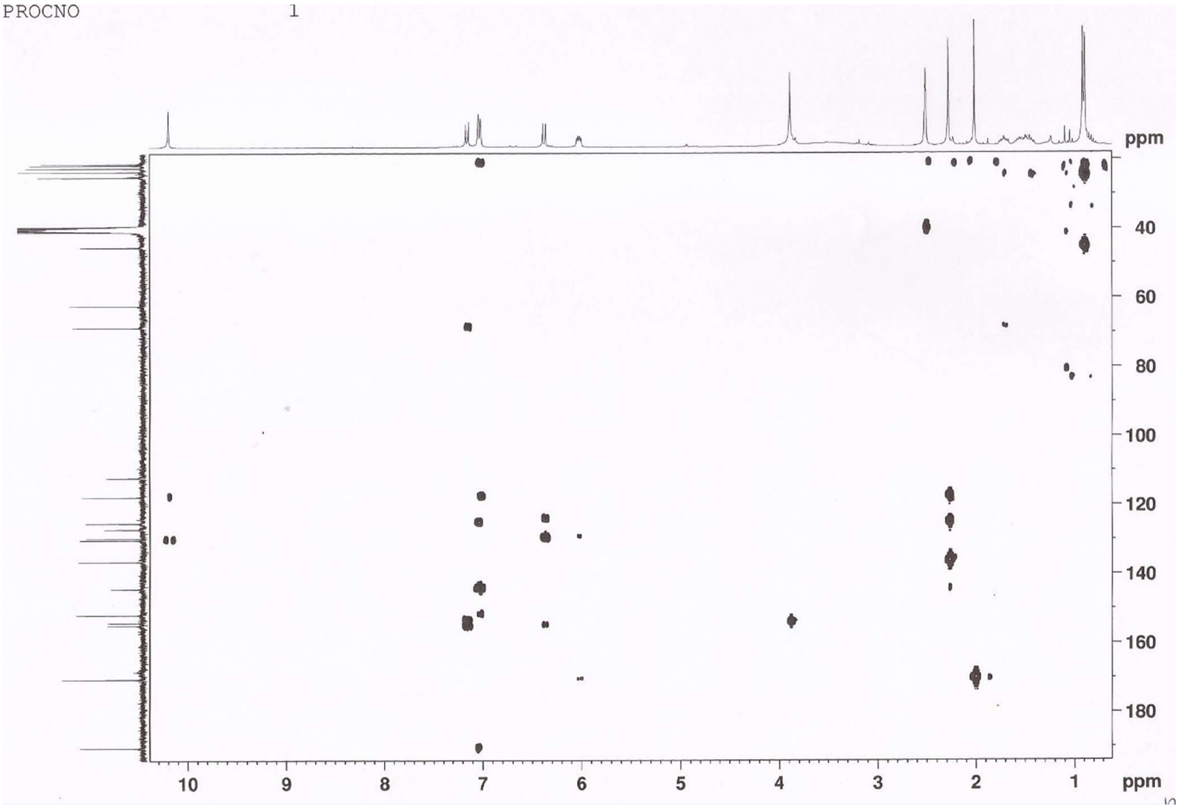

Figure S25. . (+)-HRESIMS of **5**.

Elemental Composition Report [MH]+

Single Mass Analysis

Tolerance = 5.0 PPM / DBE: min = -1.5, max = 50.0

Element prediction: Off

Number of isotope peaks used for i-FIT = 3

Monoisotopic Mass, Even Electron Ions

26 formula(e) evaluated with 1 results within limits (up to 100 best isotopic matches for each mass)

Elements Used:

C: 23-23 H: 0-150 O: 0-30

Minimum:				-1.5				
Maximum:		5.0	5.0	50.0				
Mass	Calc. Mass	mDa	PPM	DBE	i-FIT	Norm	Conf(%)	Formula
431.1700	431.1706	-0.6	-1.4	10.5	775.4	n/a	n/a	C23 H27 O8

Elemental Composition Report [MNa]+

Single Mass Analysis

Tolerance = 5.0 PPM / DBE: min = -1.5, max = 50.0

Element prediction: Off

Number of isotope peaks used for i-FIT = 3

Monoisotopic Mass, Even Electron Ions

55 formula(e) evaluated with 1 results within limits (up to 100 best isotopic matches for each mass)

Elements Used:

C: 23-23 H: 0-150 O: 0-30 Na: 0-1

Minimum:				-1.5				
Maximum:		5.0	5.0	50.0				
Mass	Calc. Mass	mDa	PPM	DBE	i-FIT	Norm	Conf(%)	Formula
453.1522	453.1525	-0.3	-0.7	10.5	543.3	n/a	n/a	C23 H26 O8 Na

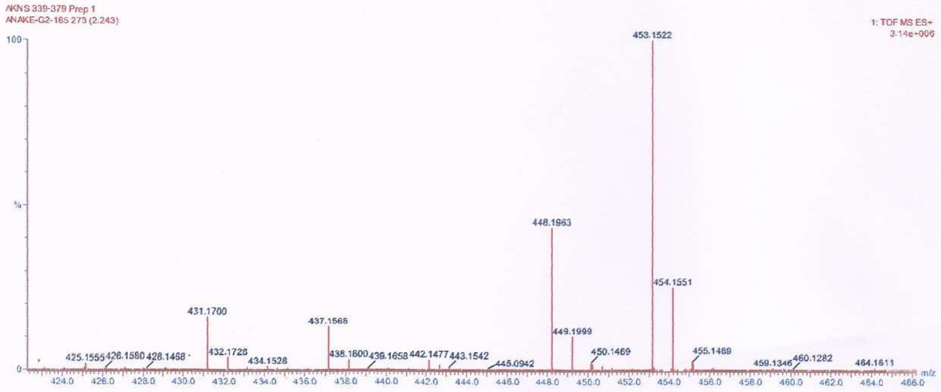

Figure S26. ^{1}H NMR spectrum of **6** (DMSO-d_6, 300 MHz).

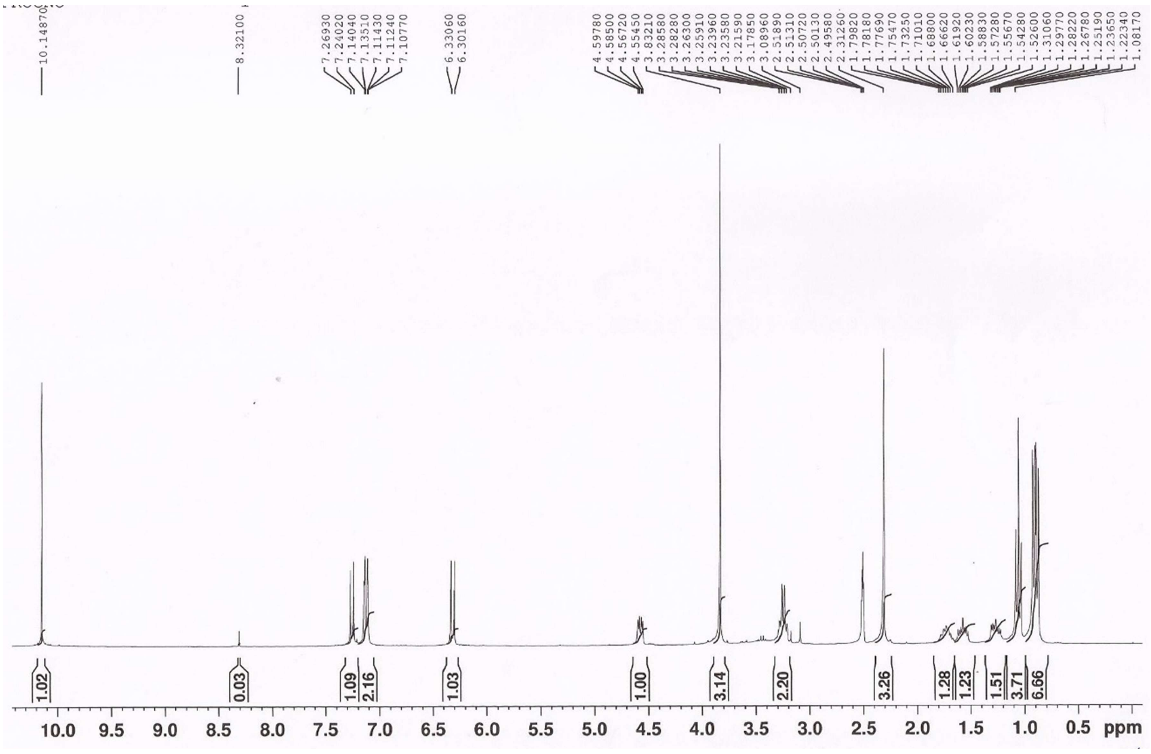

Figure S27. ^{13}C NMR spectrum of **6** (DMSO-d_6, 300 MHz).

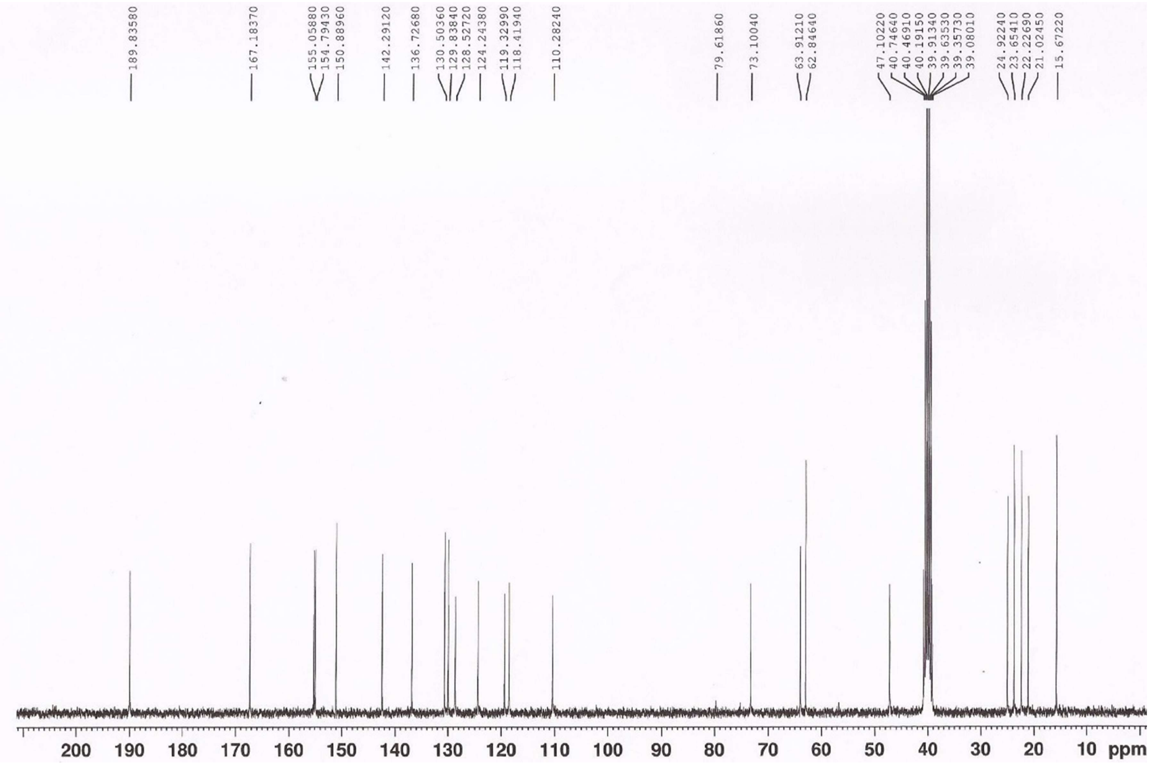

Figure S28. COSY spectrum of **6** (DMSO-d_6, 300 MHz).

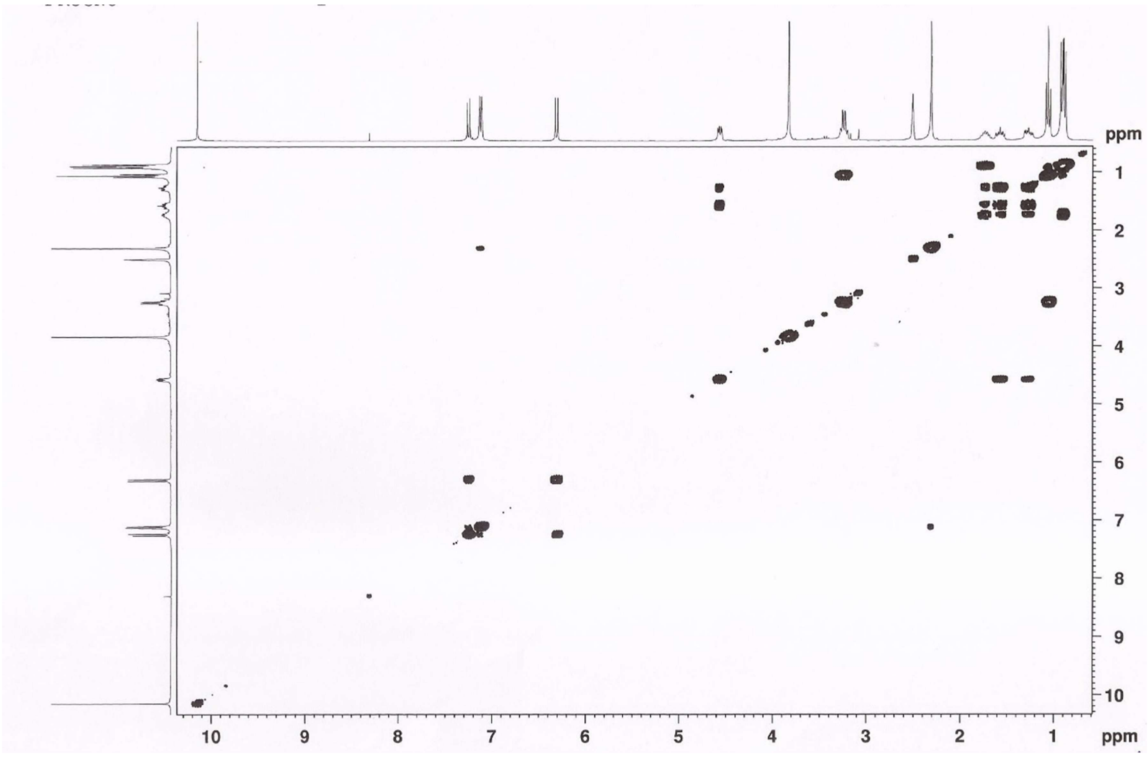

Figure S29. HSQC spectrum of **6** (DMSO-d_6, 300 MHz).

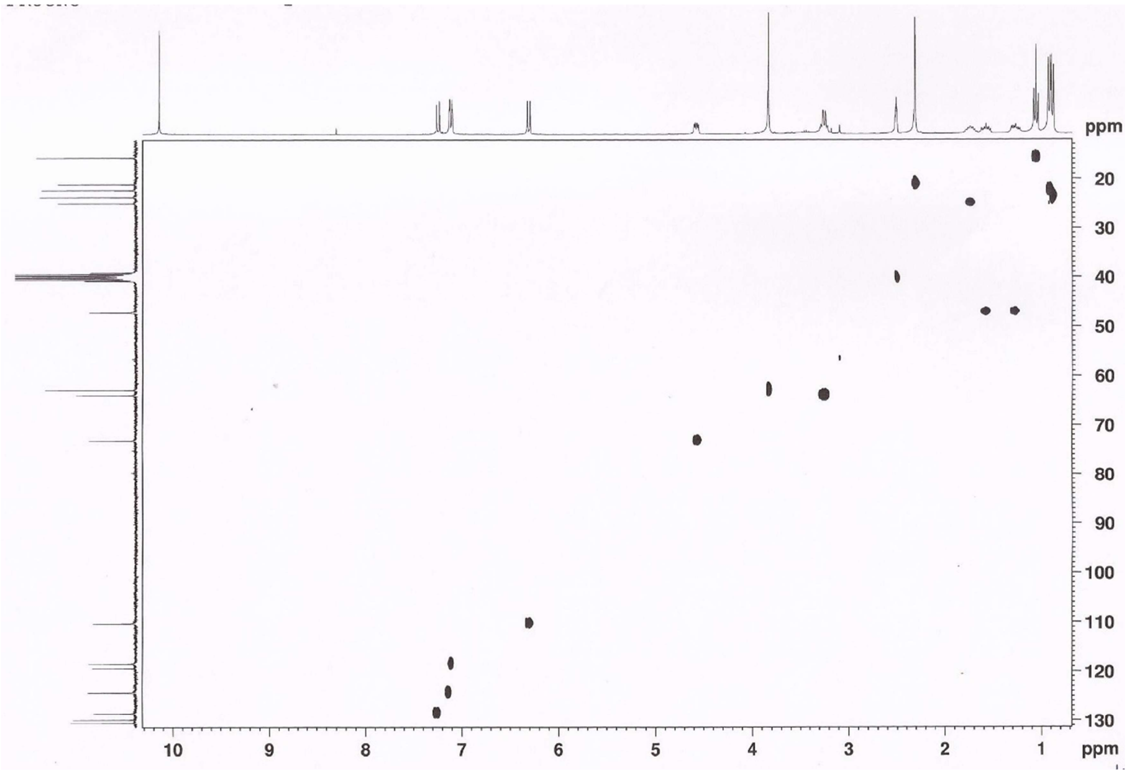

Figure S30. HMBC spectrum of **6** (DMSO-d_6, 300 MHz).

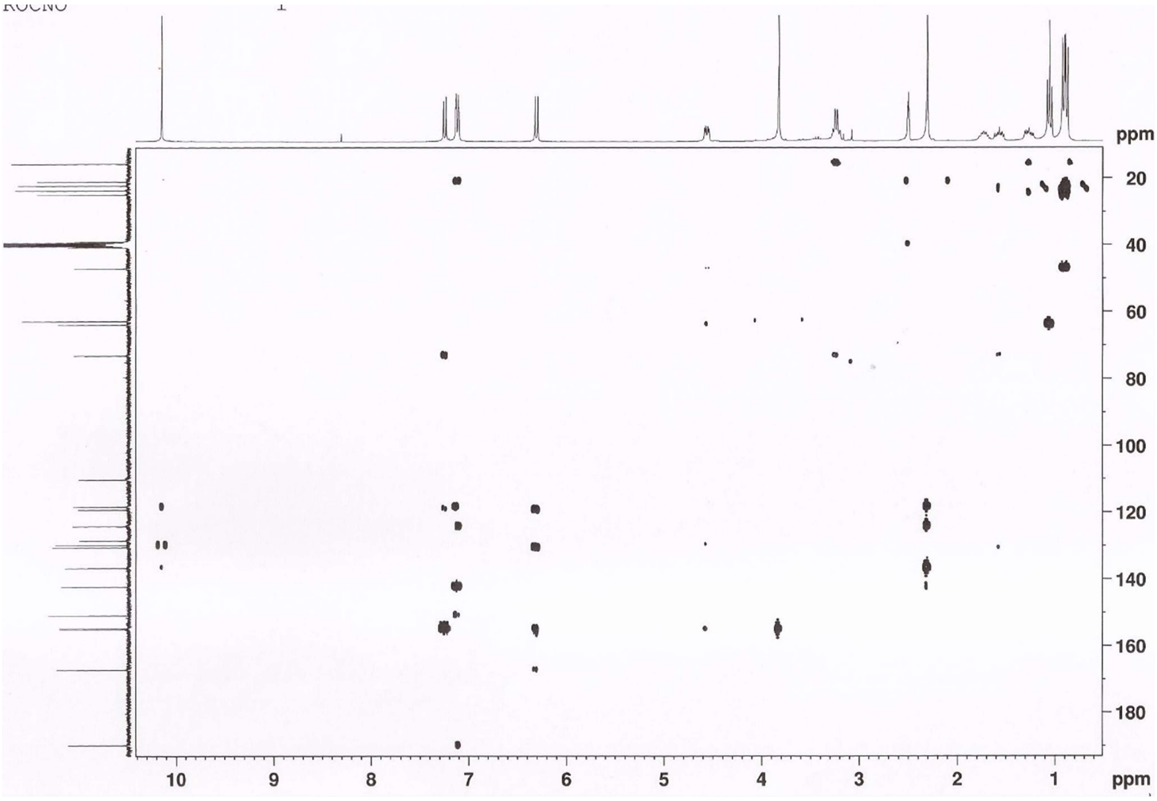

Figure S31. (+)-HRESIMS of **6**.

Elemental Composition Report [MH]+

Single Mass Analysis
Tolerance = 5.0 PPM / DBE: min = -1.5, max = 50.0
Element prediction: Off
Number of isotope peaks used for i-FIT = 3
Monoisotopic Mass, Even Electron Ions
26 formula(e) evaluated with 1 results within limits (up to 100 best isotopic matches for each mass)
Elements Used:
C: 23-23 H: 0-150 O: 0-30

Minimum:				-1.5				
Maximum:		5.0	5.0	50.0				
Mass	Calc. Mass	mDa	PPM	DBE	i-FIT	Norm	Conf(%)	Formula
417.1915	417.1913	0.2	0.5	9.5	377.3	n/a	n/a	C23 H29 O7

Elemental Composition Report [MNa]+

Single Mass Analysis
Tolerance = 5.0 PPM / DBE: min = -1.5, max = 50.0
Element prediction: Off
Number of isotope peaks used for i-FIT = 3
Monoisotopic Mass, Even Electron Ions
54 formula(e) evaluated with 1 results within limits (up to 100 best isotopic matches for each mass)
Elements Used:
C: 23-23 H: 0-150 O: 0-30 Na: 0-1

Minimum:				-1.5				
Maximum:		5.0	5.0	50.0				
Mass	Calc. Mass	mDa	PPM	DBE	i-FIT	Norm	Conf(%)	Formula
439.1735	439.1733	0.2	0.5	9.5	541.9	n/a	n/a	C23 H28 O7 Na

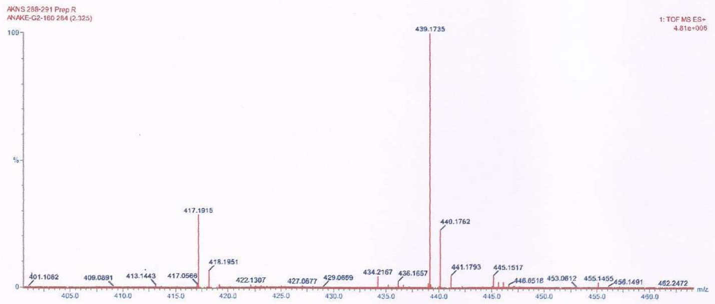

Figure S32. ^{1}H NMR spectrum of **7** (DMSO-d_6, 500 MHz).

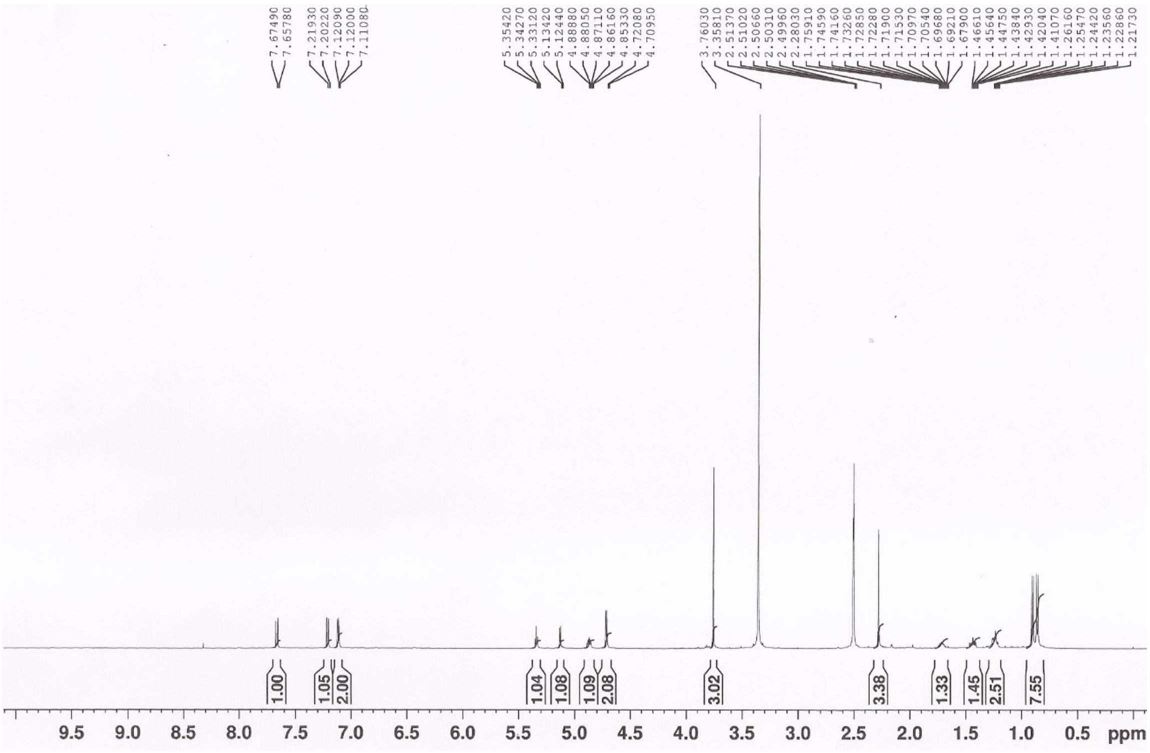

Figure S33. ^{13}C NMR spectrum of **7** (DMSO-d_6, 125 MHz).

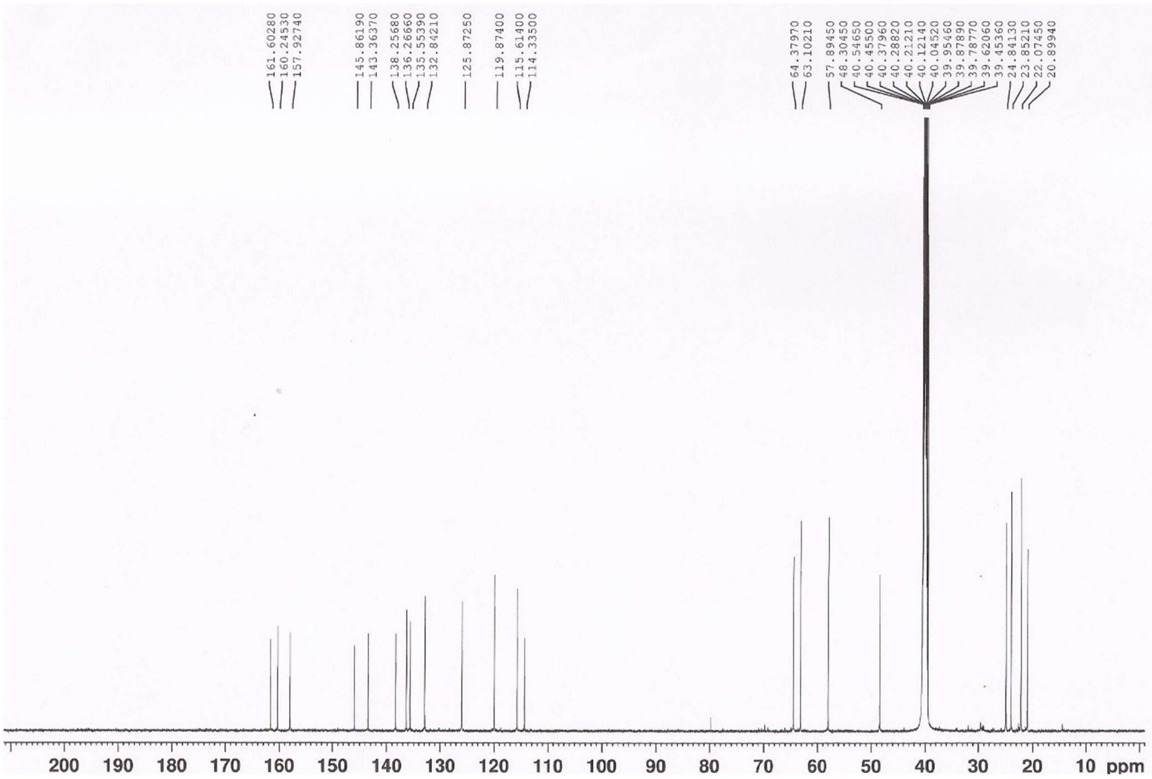

Figure S34. COSY spectrum of **7** (DMSO-d_6, 500 MHz).

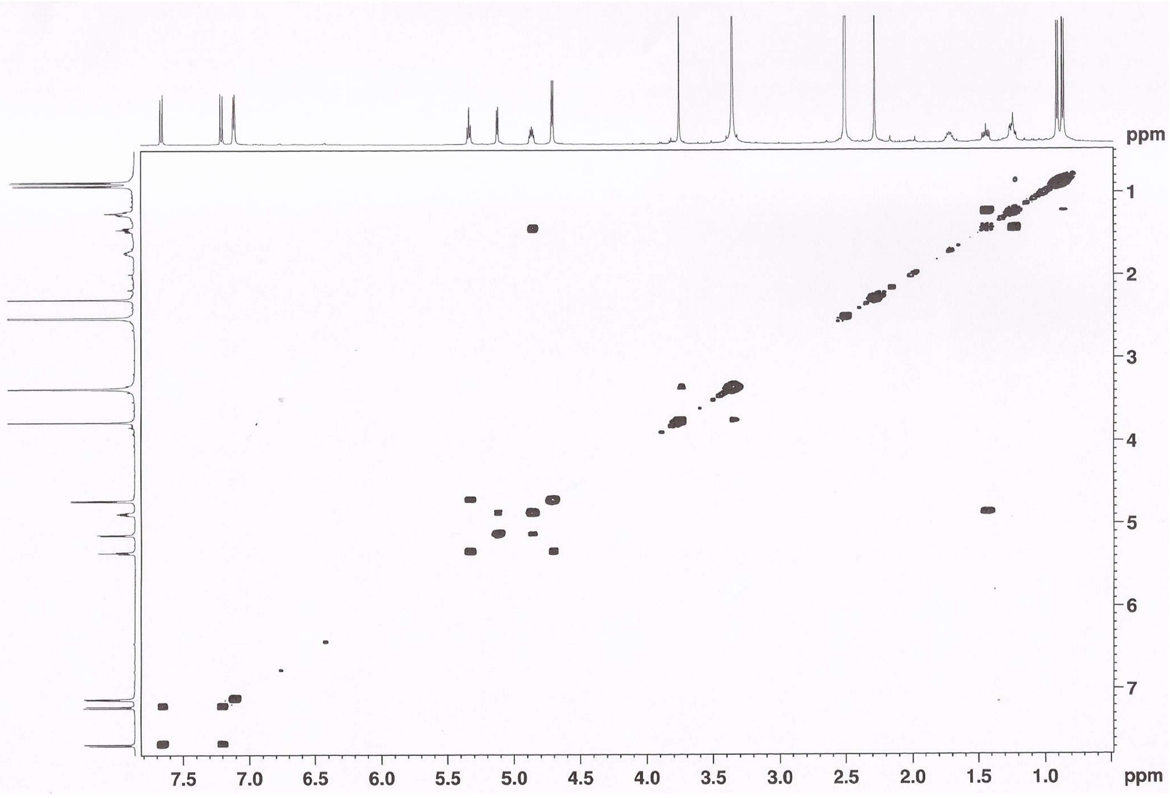

Figure S35. HSQC spectrum of **7** (DMSO-d_6, 500 MHz).

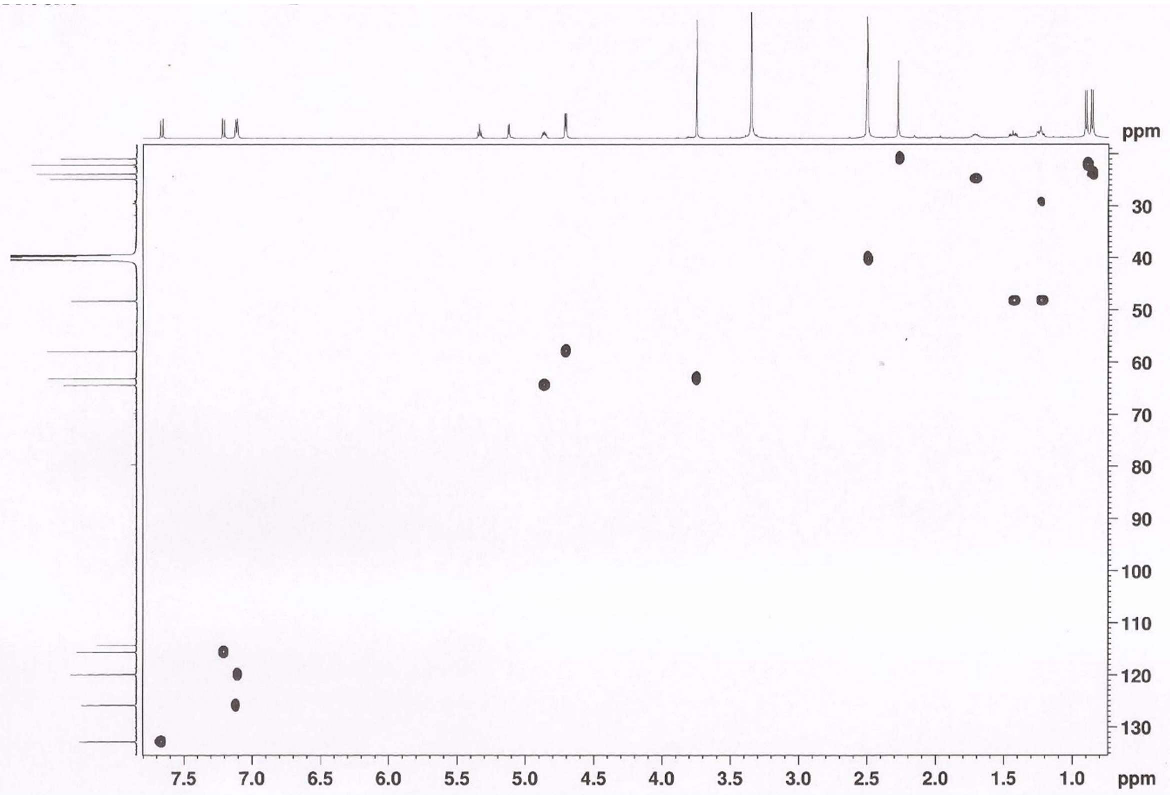

Figure S36. HMBC spectrum of **7** (DMSO-d_6, 500 MHz).

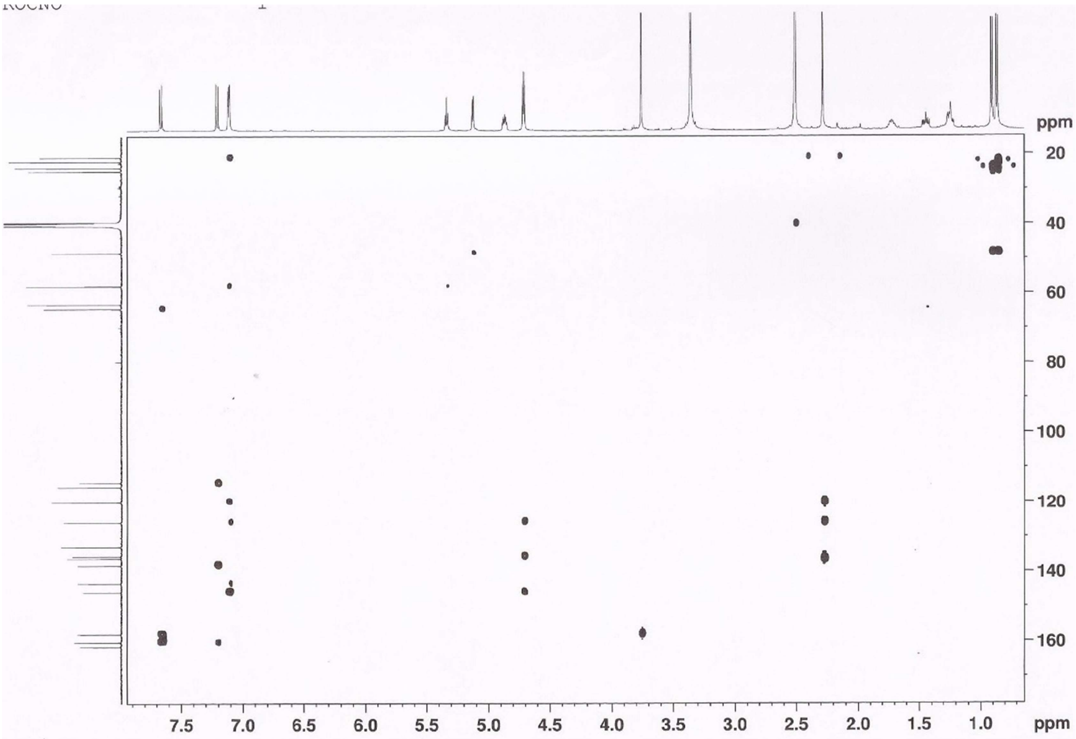

Figure S37. (+)-HRESIMS of **7**.

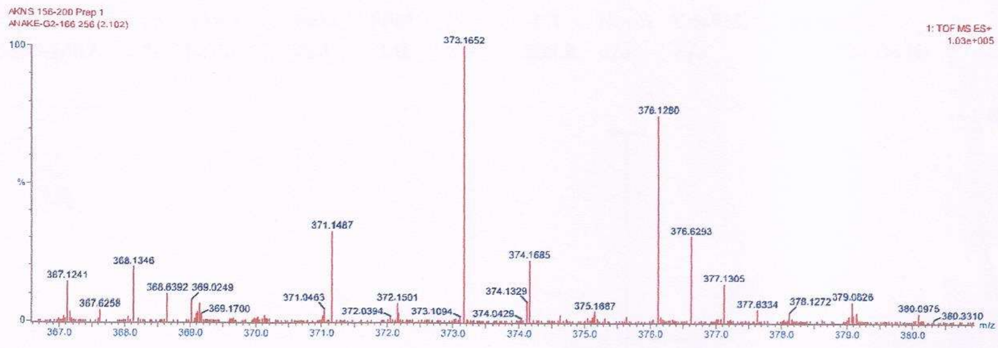

Elemental Composition Report [MH]+

Single Mass Analysis
Tolerance = 5.0 PPM / DBE: min = -1.5, max = 50.0
Element prediction: Off
Number of isotope peaks used for i-FIT = 3
Monoisotopic Mass, Even Electron Ions
24 formula(e) evaluated with 1 results within limits (up to 100 best isotopic matches for each mass)
Elements Used:
C: 21-21 H: 0-150 O: 0-30

Minimum:				-1.5				
Maximum:		5.0	5.0	50.0				
Mass	Calc. Mass	mDa	PPM	DBE	i-FIT	Norm	Conf(%)	Formula
373.1652	373.1651	0.1	0.3	9.5	685.0	n/a	n/a	C21 H25 O6

Figure S38. ^{1}H NMR spectrum of **8** (DMSO-d_6, 300 MHz).

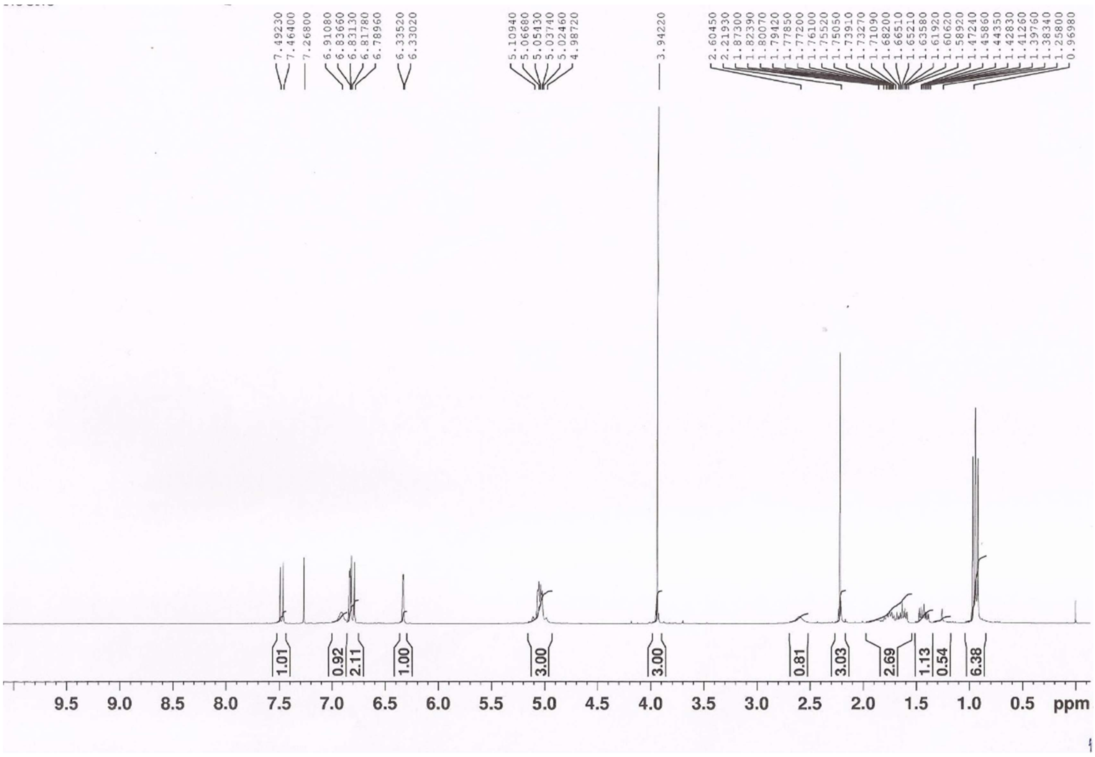

Figure S39. ^{13}C NMR spectrum of **8** (DMSO-*d*$_6$, 75 MHz).

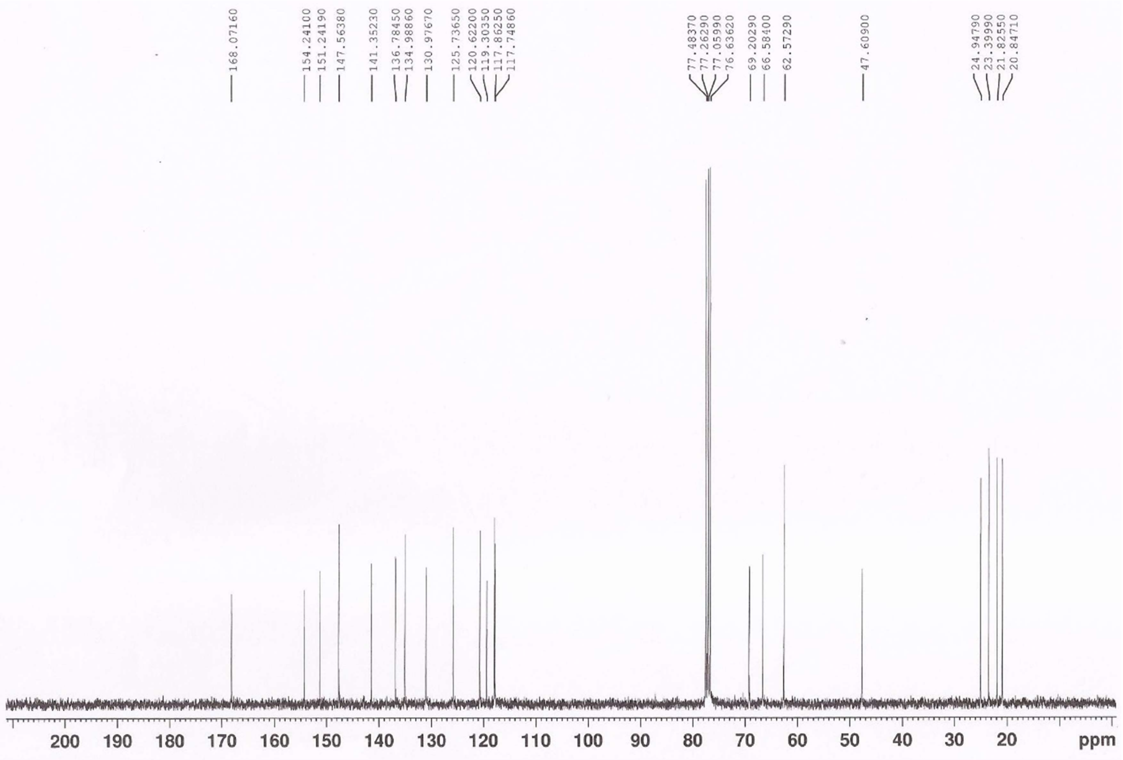

Figure S40. COSY spectrum of **8** (DMSO-*d*$_6$, 300 MHz).

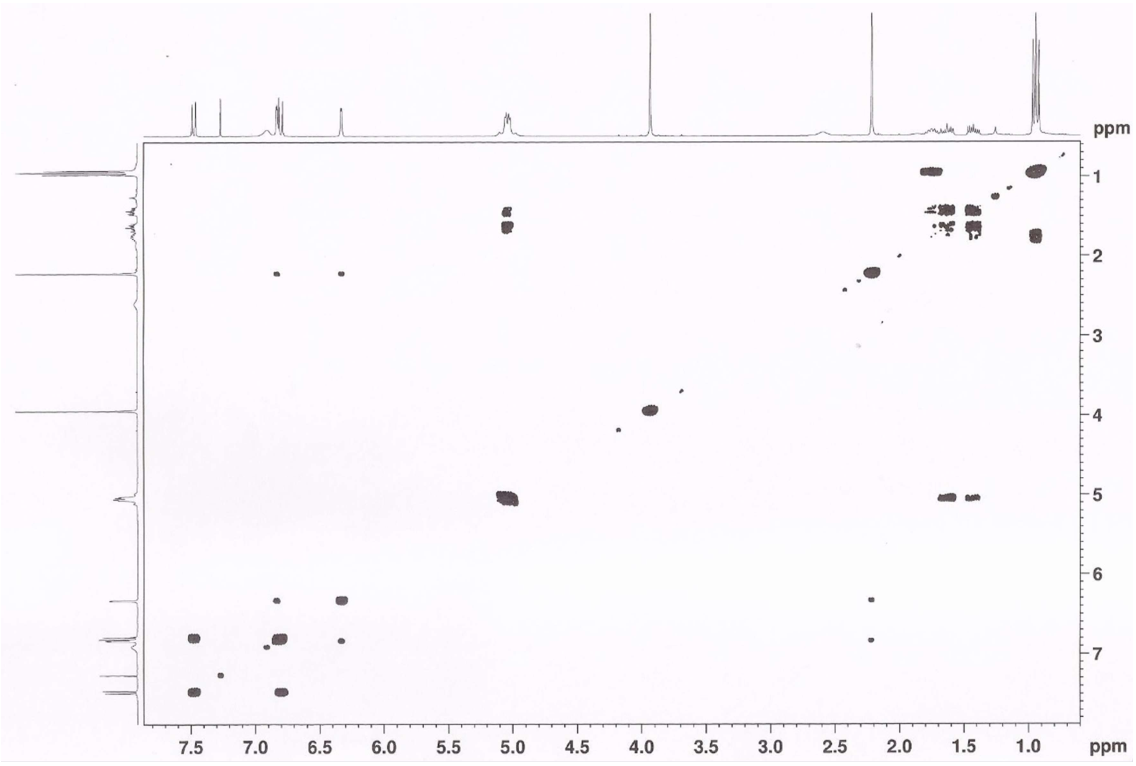

Figure S41. HSQC spectrum of **8** (DMSO-*d*$_6$, 300 MHz).

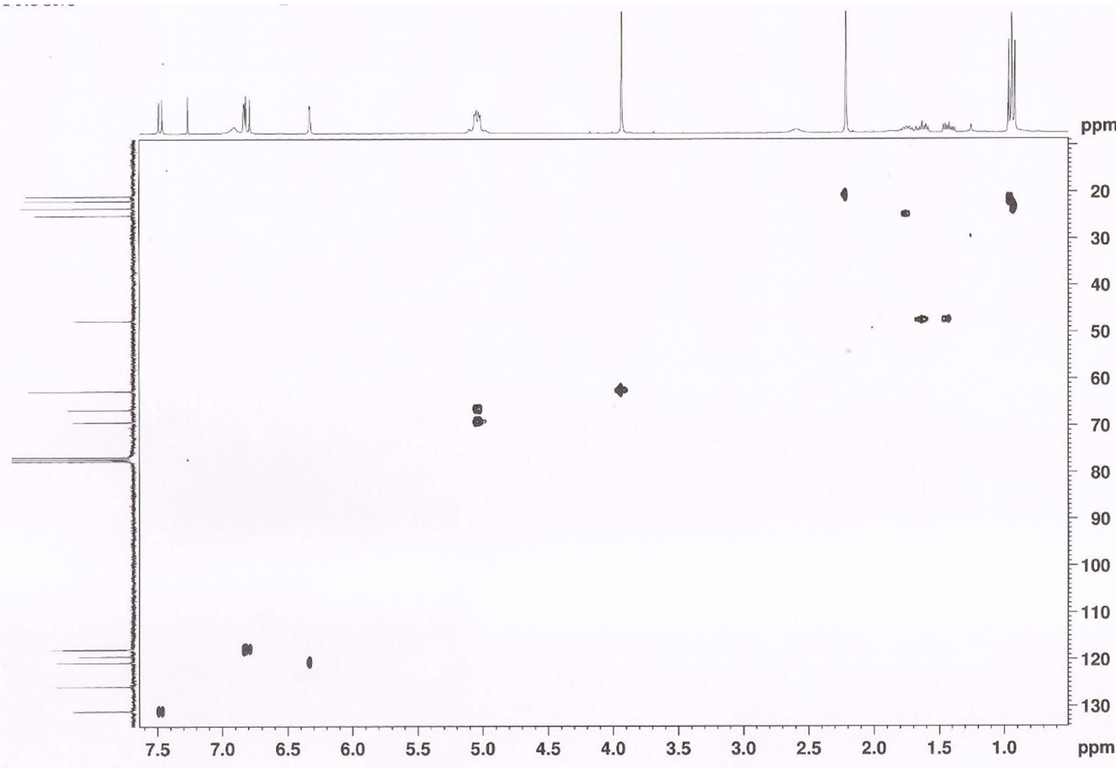

Figure S42. HMBC spectrum of **8** (DMSO-*d*$_6$, 300 MHz).

Figure S43. (+)-HRESIMS of **8**.

Elemental Composition Report [MH]+

Single Mass Analysis
Tolerance = 5.0 PPM / DBE: min = -1.5, max = 50.0
Element prediction: Off
Number of isotope peaks used for i-FIT = 3
Monoisotopic Mass, Even Electron Ions
24 formula(e) evaluated with 1 results within limits (up to 100 best isotopic matches for each mass)
Elements Used:
C: 21-21 H: 0-150 O: 0-30

Minimum:				-1.5				
Maximum:		5.0	5.0	50.0				
Mass	Calc. Mass	mDa	PPM	DBE	i-FIT	Norm	Conf(%)	Formula
373.1654	373.1651	0.3	0.8	9.5	768.4	n/a	n/a	C21 H25 O6

Elemental Composition Report [MNa]+

Single Mass Analysis
Tolerance = 5.0 PPM / DBE: min = -1.5, max = 50.0
Element prediction: Off
Number of isotope peaks used for i-FIT = 3
Monoisotopic Mass, Even Electron Ions
49 formula(e) evaluated with 1 results within limits (up to 100 best isotopic matches for each mass)
Elements Used:
C: 21-21 H: 0-150 O: 0-30 Na: 0-1

Minimum:				-1.5				
Maximum:		5.0	5.0	50.0				
Mass	Calc. Mass	mDa	PPM	DBE	i-FIT	Norm	Conf(%)	Formula
395.1473	395.1471	0.2	0.5	9.5	822.8	n/a	n/a	C21 H24 O6 Na

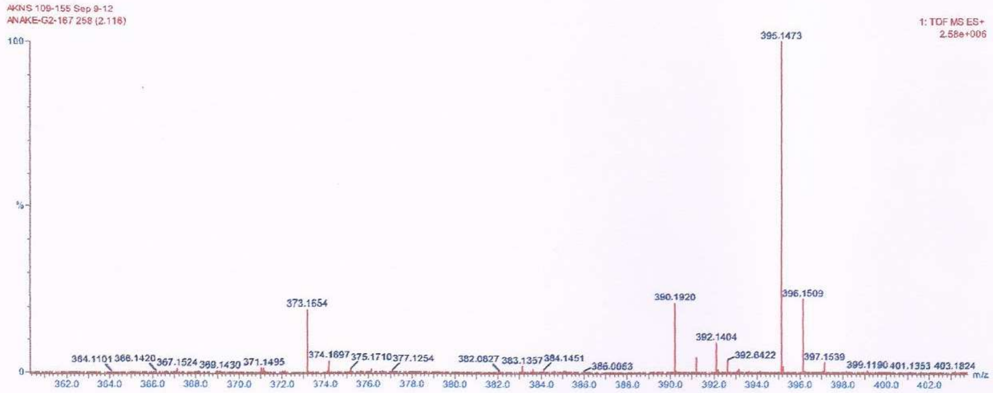

Table S1. ^{1}H and ^{13}C NMR (DMSO-d_6, 300 and 75 MHz) and HMBC assignment for **1**.

Position	δ_C, type	δ_H, *J* in Hz	COSY	HMBC
1	169.9, C	-		
3	75.8, CH	4.69, m	H_2-4, Me-9	
4	34.2, CH_2	2.80, dd (16.5, 11.1)<br>2.93 dd (16.5, 3.5)	H-5	C-3, 4a, 5, 8a, 9
4a	142.7, C	-		
5	107.2, CH	6.24, brs	H_2-1’	C-4,7,8a
6	164.9, C	-		
7	101.3, CH	6.19, d (1.9)		C-5, 6,8a
8	163.9, C	-		
8a	100.5, C			
9	20.7, CH_3	1.39, d (6.3)	H-3	C-3, 4
OH-8	-	11.13, s		C-7, 8

Table S2. ^{1}H and ^{13}C NMR (DMSO-d_6, 300 and 75 MHz) and HMBC assignment for **2**.

Position	δ_C, type	δ_H, *J* in Hz	COSY	HMBC
1	161.9, C			
2	125.2 CH	7.13, d (1.6)	H-4	C-1, 1’, 4, 9a
3	148.8, C			
4	120.0, CH	7.46, d (1.6)	H-2	C, 1’, 2, 9a, 10
4a	132.2, C			
5	107.8, CH	7.18, d (2.3)	H-7	C-7, 8a, 10
6	165.7, C			
7	105.4, CH	6.08, d (2.3)	H-5	C-5, 6, 8a
8	164.0, C			
8a	112.6, C			
9	186.6, CO			
9a	115.1, C			
10	183.0, CO			
10a	137.3, C			
OMe-8	56.7, CH_3	3.89,s		C-8
1’	45.5, CH_2	2.71, m	H-2’	C-2’, 3
2’	67.1, CH	3.86, m	H_2-1’, H_3-3’	C-3
3’	23.9, CH_3	1.09, d (6.1)	H-2’	C-1’, 2’
OH-1	-	13.30, s		C-1, 2, 9a

Table S3. ^{1}H and ^{13}C NMR (DMSO-d_6, 500 and 125 MHz) and HMBC assignment for **3**.

Position	δ_C, type	δ_H, *J* in Hz	COSY	HMBC
1	162.1, C			
2	122.8 CH	7.25, d (1.8)	H-4	C1’, 4, 9a
3	144.5, C			

4	117.0, CH	7.55, d (1.8)	H-2	C-2, 1', 9a, 10
4a	133.0, C			
5	108.7, CH	7.16, d (2.2)	H-7	C-7. 8a, 10
6	166.6, C			
7	105.6, CH	6.76, d (2.2)	H-5	C-5, 6, 8a
8	164.2, C			
8a	112.2, C			
9	186.1, CO			
9a	116.5, C			
10	182.9, CO			
10a	137.1, C			
OMe-8	56.6, CH_3	3.89, s		C-8
1'	64.8, CH_2	5.16, s		C-2, 3, 4, CO (Ac)
CO (Ac)	170.7, CO			
Me (Ac)	21.1, CH_2	2.13, s		CO (Ac)
OH-1	-	13.52, s		

Table S4. ^{1}H and ^{13}C NMR (300 and 75 MHz, DMSO-d_6) and HMBC assignment of **5**.

Position	δ_C, type	δ_H, *J* in Hz	COSY	HMBC
1	124.0, C			
2	153.9, C			
3	129.4, C			
4	126.8, CH	7.16, d (8.6)	H-5	C-2, 6, 8
5	112.2, CH	6.38, d (8.6)	H-4	C-1, 3, 6
6	154.7, C			
7	168.2, CO			
8	68.4, CH	4.02, dd (9.0, 4.5)	H-9	C-3, CO (Ac)
9a<br>b	45.3, CH_2	1.48, m<br>1.70, m	H-8, 10	C-8, 10
10	24.9, CH	1.56, m	H-9	
11	23.3, CH_3	0.90, d (6.2)	H-10	C-9, 10, 12
12	22.2, CH_3	0.90, d (6.2)	H-10	C-9, 10, 11
1'	130.0, C			
2'	144.1, C			
3'	151.7, C			
4'	125.1, CH	7.02, d (1.3)	H-6', 8'	C-2', 3', 6', 8'
5'	136.3, C			
6'	117.6, CH	7.05, d (1.3)	H-4', 8'	C-2', 3'(w), 4', 7', 8'
7'	190.3, COH	10.27, s		C-1', 6'
8'	21.1, CH_3	2.27, s		C-4', 5', 6'
OMe-2	62.1, OCH_3	3.88, s		C-2
CO (Ac)	170.3, CO			
Me (Ac)	21.4, CH_3	2.00, s		CO (Ac)

w= weak

Table S5. ^{1}H and ^{13}C NMR (300 and 75 MHz, $CDCl_3$) and HMBC assignment of **8**.

Position	δ_C, type	δ_H, J in Hz	COSY	HMBC
1	125.7, C			
2	154.2, C			
3	136.8, C			
4	131.0, CH	7.48, d (8.5)	H-5	C-1 (w), 2, 6, 8
5	117.7, CH	6.80, d (8.5)	H-4	C-1, 3, 6, 7 (w)
6	151.2, C			
7	168.0, CO			
8	66.6, CH	5.04, m	H-9a, 9b	C-2, 3, 9
9a<br>b	47.6, CH_2	1.43, ddd (13.7, 8.9, 4.2)<br>1.64, ddd (13.7, 8.9, 5.1)	H-8	C-3, 8, 11
10	24.9, CH	1.76, m	H-12, 13	
11	23.4, CH_3	0.93, d (6.7)	H-10	C-9, 10, 12
12	21.8, CH_3	0.96, d (6.7)	H-10	C-9, 10, 11
1’	125.7, C			
2’	141.4, C			
3’	147.6, C			
4’	117.9, CH	6.83, d (1.5)	H-6’	C-2’, 3’, 6’, 8’
5’	135.0, C			
6’	120.6, CH	6.33, d (1.5)	H-4’	C-2’, 4’, 7’, 8’
7’	69.2 CH_2	5.04, m		
8’	20.8, CH_3	2.22, s		C-4’, 5’, 6’
OMe-2	62.6, OCH_3	3.94, s		C-2
OH-3’		6.91, br		
